# Structural insights into Semiliki forest virus receptor binding modes indicate novel mechanism of virus endocytosis

**DOI:** 10.1371/journal.ppat.1012770

**Published:** 2024-12-20

**Authors:** Decheng Yang, Nan Wang, Bingchen Du, Zhenzhao Sun, Shida Wang, Xijun He, Jinyue Wang, Tao Zheng, Yutao Chen, Xiangxi Wang, Jingfei Wang

**Affiliations:** 1 State Key Laboratory for Animal Disease Control and Prevention & National Data Center for Animal Infectious Diseases, Harbin Veterinary Research Institute, Chinese Academy of Agricultural Sciences, Harbin, People’s Republic of China; 2 CAS Key Laboratory of Infection and Immunity, National Laboratory of Macromolecules, Institute of Biophysics, Chinese Academy of Sciences, Beijing, China; 3 University of Chinese Academy of Sciences, Beijing, China; Institut Pasteur, FRANCE

## Abstract

The Very Low-Density Lipoprotein Receptor (VLDLR) is an entry receptor for the prototypic alphavirus Semliki Forest Virus (SFV). However, the precise mechanisms underlying the entry of SFV into cells mediated by VLDLR remain unclear. In this study, we found that of the eight class A (LA) repeats of the VLDLR, only LA2, LA3, and LA5 specifically bind to the native SFV virion while synergistically promoting SFV cell attachment and entry. Furthermore, the multiple cryo-electron microscopy structures of VLDLR-SFV complexes and mutagenesis studies have demonstrated that under physiological conditions, VLDLR primarily binds to E1-DIII of site-1, site-2, and site-1’ at the twofold symmetry axes of SFV virion through LA2, LA3, and LA5, respectively. These findings unveil a novel mechanism for viral entry mediated by receptors, suggesting that conformational transitions in VLDLR induced by multivalent binding of LAs facilitate cellular internalization of SFV, with significant implications for the design of antiviral therapeutics.

## Author summary

Alphaviruses are enveloped, arthropod-transmitted RNA viruses that are important emerging pathogens causing human disease worldwide. Semliki Forest Virus (SFV), a prototypical member of the genus, utilizes the very low-density lipoprotein receptor (VLDLR) as a receptor during infection in both vertebrate and invertebrate hosts. The VLDLR ectodomain contains eight analogous class A repeats (LA1-8) that interact with SFV to mediate viral entry. However, the coordination among these LAs in this process remains elusive. To elucidate the molecular basis of the SFV-VLDLR interaction, we have determined the atomic structures of the SFV particle in complex with LA3, LA5, LA3-5, or the full-length VLDLR (LA1-8) using cryo-electron microscopy. Combined with mutagenesis studies, the resulting high-resolution density maps elucidate the molecular mechanism of SFV-VLDLR engagement and viral infection. Our findings provide detailed insights into the biology of SFV entry with high-resolution details that may facilitate the rational design of novel therapeutics targeting this binding site.

## Introduction

Alphaviruses are enveloped, positive-sense, single-stranded RNA viruses in the *Togaviridae* family that are transmitted between mosquito vectors and vertebrate hosts, posing an emerging and reemerging public health threat worldwide [1]. Members of the Alphavirus genus have historically been categorized into New World and Old World viruses based on geographic origins. New World alphaviruses, such as eastern equine encephalitis virus (EEEV), western equine encephalitis virus (WEEV), and Venezuelan equine encephalitis virus (VEEV), can infect nerve cells and lead to neurological diseases with case fatality rates ranging from 30–75% for EEEV [2], 3–7% for WEEV [3] and <1% for VEEV infections [4]. Survivors often suffer from permanent and debilitating neurological complications. In contrast, diseases caused by Old World alphaviruses, such as chikungunya virus (CHIKV), Ross River virus (RRV), o’nyong’nyong (ONNV), Semliki Forest virus (SFV) and Sindbis virus (SINV), are characterized by fever, rash, myalgia, and arthralgia. Although the infections are rarely fatal, symptoms can persist for months to years [5]. Members of the Old World alphaviruses rarely cause neurological disease, but there is some evidence that SFV can cause fatal encephalitis in humans [6]. Currently, there are no drugs approved for the treatment of infections caused by alphaviruses.

The mature alphavirus virion is approximately 70 nanometers in diameter and has T = 4 icosahedral symmetry [7–9]. Trimers of E1-E2 heterodimers are organized into 80 trimeric spikes on the virion surface. E1 and E2 glycoproteins play a key role in the entry of the virus into host cells by endocytosis. While both E1 and E2 have been shown to contribute to receptor engagement [10–13], E1 also mediates membrane fusion after viral entry [14]. E1 is a class II membrane fusion protein and consists of three domains: Domain I (DI) is located at the center, DII is located at the distal end, and DIII is located close to the viral membrane [14,15]. The hydrophobic fusion loop (FL) is located at the tip of DII. E2 comprises three domains, A, B, and C. The domain A is located centrally on the surface of the spike and possesses the putative receptor binding site; the B domain is located on the distal end of the spike covering FL on E1; and the C domain is positioned close to E1 and the viral membrane [16–18]. E1 lies at the base of the trimeric spike. E2 is positioned above E1, shielding the functionally critical FL from premature exposure. E3 is a small, cysteine-rich glycoprotein that mediates the proper folding of p62 (E2 precursor) and the formation of the p62-E1 heterodimer during spike assembly [19,20]. It is cleaved by furin proteases during the maturation process in the trans-Golgi network [21].

Recognition of and interactions with cellular receptors is a critical early step in the initiation of alphavirus infection. Matrix remodeling associated 8 (MXRA8) has been identified as a receptor for many Old World alphaviruses (CHIKV, RRV, MAYV, and ONNV) [22–24], the evolutionarily conserved low-density lipoprotein receptor (LDLR) could act as an entry factor for multiple alphaviruses (EEEV, WEEV, RRV and SFV) [25–27], and LDLR class A domain-containing 3 (LDLRAD3) has been identified as the receptor specifically for VEEV [28]. The high-resolution structural analysis of virus-receptor complexes (MXRA8-CHIKV and LDLRAD3-VEEV) has shown that both MXRA8 and LDLRAD3 bind their respective viruses in clefts formed between neighboring E1-E2 heterodimers of the trimeric spikes [10–13]. A recent study reported that the very low-density lipoprotein receptor (VLDLR) is used as a functional receptor by multiple alphaviruses, including SFV, EEEV, and SINV [29]. The ligand-binding domain (LBD), composed of eight LDLR type A (LA) repeats, is necessary and sufficient for mediating infection. EEEV utilizes multiple distinct sites on the E2 glycoprotein to interact with VLDLR [30–32], in contrast to SFV which employs a single site on the E1 glycoprotein (E1-DIII) to bind to VLDLR LA repeats [33,34]. Unfortunately, since the densities of multiple similar LA repeats bound with SFV E1-DIII are smeared in the previously reported cryo-electron microscopy (cryo-EM) structures [33], the detailed recognition mechanism of VLDLR by SFV remains unclear. To elucidate the molecular mechanism of the SFV-VLDLR interaction, we performed detailed functional studies and characterized multiple cryo-EM structures of full-length VLDLR and individual LA domains in complex with the native SFV virion. Combined with the mutagenesis study, our structures dissect how SFV engages its receptor VLDLR to facilitate attachment and infection of cells.

## Results

### VLDLR-mediated cell entry of SFV through the LA2, LA3, and LA5 domains

**Fig 1:**
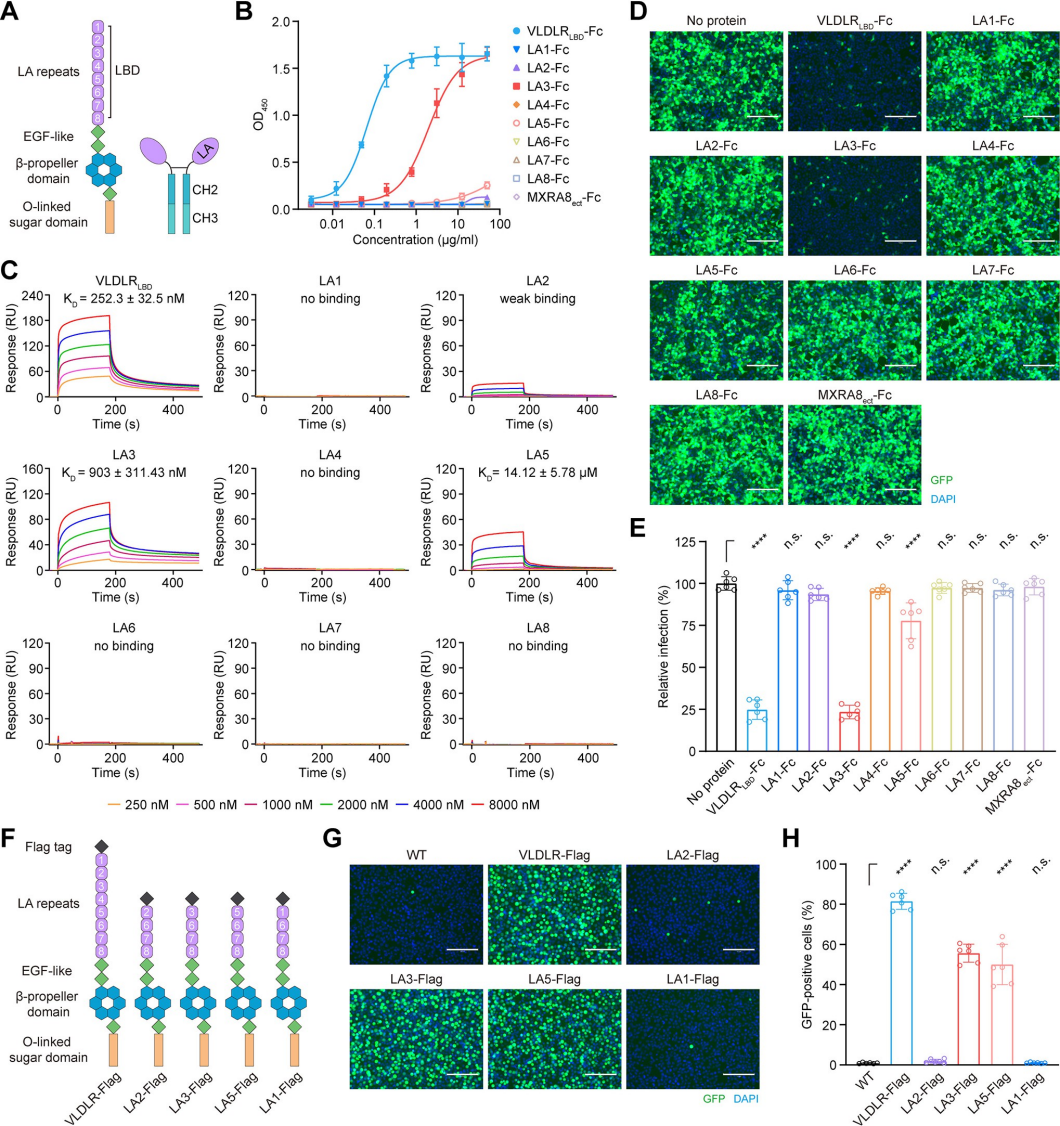
Individual LA3 or LA5 domains of VLDLR LBD can mediate cell attachment and entry of SFV. (**A**) Schematic of the ectodomain of VLDLR (left) and LA repeats-Fc fusions (right). LA repeats of VLDLR LBD are numbered. (**B**) Binding of different LA repeats-Fc fusions, positive control (VLDLR_LBD_-Fc) or negative control (MXRA8_ect_-Fc) to SFV virions measured by enzyme-linked immunosorbent assay (ELISA). Mean ± SD of three experiments (n = 6). OD_450_, optical density at 450 nm. (**C**) Specific interactions between VLDLR_LBD_, or different LA repeats and SFV were characterized by SPR. SFV virions were immobilized on the CM5 chip using standard amine coupling chemistry and were then tested for binding with gradient concentrations of VLDLR_LBD_-Fc or different LA repeats-Fc fusion proteins. The data were analyzed with Biacore 8K Evaluation software (GE Healthcare). K_D_ values are shown as mean ± SEM of three independent experiments. (**D**) Infection of HEK293T cells with eGFP-expressing SFV (SFV-eGFP) at a multiplicity of infection (MOI) of 5 in the presence of the indicated proteins (100 μg/mL). Cells were imaged by fluorescence microscopy: scale bar, 150 μm. The experiment was performed twice independently with similar results and representative images are shown. (**E**) Infection of HEK293T cells with SFV-eGFP in the presence of the indicated proteins (100 μg/mL), measured by FACS. Mean ± SD of three experiments (n = 6), One-way ANOVA with Tukey’s multiple comparisons test, ****P < 0.0001; n.s., not significant. (**F**) VLDLR ectodomain and deletion constructs. LBD LA repeats are numbered. (**G**) Infection of wild-type K562 cells or K562 cells expressing indicated constructs shown in (F) with SFV-eGFP. Cells were imaged by fluorescence microscopy: scale bar, 150 μm. The experiment was performed twice independently with similar results and representative images are shown. (**H**) Infection of wild-type or transduced K562 cells with SFV-eGFP measured by FACS. Mean ± SD of three experiments (n = 6), One-way ANOVA with Tukey’s multiple comparisons test, ****P < 0.0001; n.s., not significant.

VLDLR ectodomain consists of an N-terminal ligand-binding domain (LBD) followed by three epidermal growth factor (EGF)-like domains, β-propeller domains, and an O-linked sugar domain (Fig 1A, left panel). The LBD is comprised of eight cysteine-rich complement-type repeats, also known as LA domains (LA1 to LA8), which contribute to binding with SFV [29]. To determine which LA repeat(s) interact with SFV and facilitate the virus entry, we generated Fc fusion proteins of the LBD of human VLDLR (VLDLR_LBD_-Fc), each LA repeats (LA-Fc), and the MXRA8 ectodomain (MXRA8_etc_-Fc) as a control (Fig 1A, right panel). ELISA-based binding assays for SFV virion and Fc fusion protein revealed that both VLDLR_LBD_-Fc and LA3-Fc exhibited significant binding to SFV, while high concentrations of LA5-Fc and LA2-Fc also exhibited some binding potential to SFV. Conversely, other constructs such as LA1-Fc, LA4-Fc, LA6-Fc, LA7-Fc, and LA8-Fc, as well as the control MXRA8_etc_-Fc, showed no discernible binding effects (Fig 1B). The binding affinity of the Fc fusion proteins to SFV virion was also analyzed using surface plasmon resonance (SPR). Compared with the other LA repeats, VLDLR_LBD_-Fc and LA3-Fc exhibited a higher binding affinity to viral particles, with K_*D*_ values of 252.3 nM and 903.0 nM, respectively. LA5-Fc and LA2-Fc demonstrated a relatively weak binding affinity to the virion, whereas the binding of LA1-Fc, LA4-Fc, LA6-Fc, LA7-Fc, or LA8-Fc to SFV virion was undetectable by SPR (Fig 1C).

To assess the potential inhibitory effect of each LA repeat on virus infection, eGFP-expressing SFV (SFV-eGFP) was pre-incubated with each LA-Fc fusion protein before being incubated with HEK 293T cells. LA3-Fc and VLDLR_LBD_-Fc almost completely blocked the infection of the virus. LA5-Fc also significantly inhibited the viral infection compared to the control, where virions were not pre-incubated with any recombinant protein. However, LA2-Fc, control MXRA8_etc_-Fc, and other constructs (LA1-Fc, LA4-Fc, LA6-Fc, LA7-Fc, and LA8-Fc) did not prevent the viral infection (Fig 1D and 1E). In addition, the support of SFV infection by individual LA repeat was assessed by ectopically expressing VLDLR truncation mutants, including VLDLR-Flag, LA1-Flag, LA2-Flag, LA3-Flag, and LA5-Flag (Fig 1F), in cell line K562 which is non-permissive for SFV. Surface expression of VLDLR variants was confirmed using an N-terminal Flag tag placed downstream of the signal peptide (S1A Fig). LA3 and LA5, as the full-length VLDLR, rendered K562 cells highly susceptible to SFV infection, but LA1 and LA2 did not. This is consistent with the results of viral infection-blocking assays (Figs 1G, 1H and S1B).

### Overall structures of single LA domain in complex with SFV

**Fig 2:**
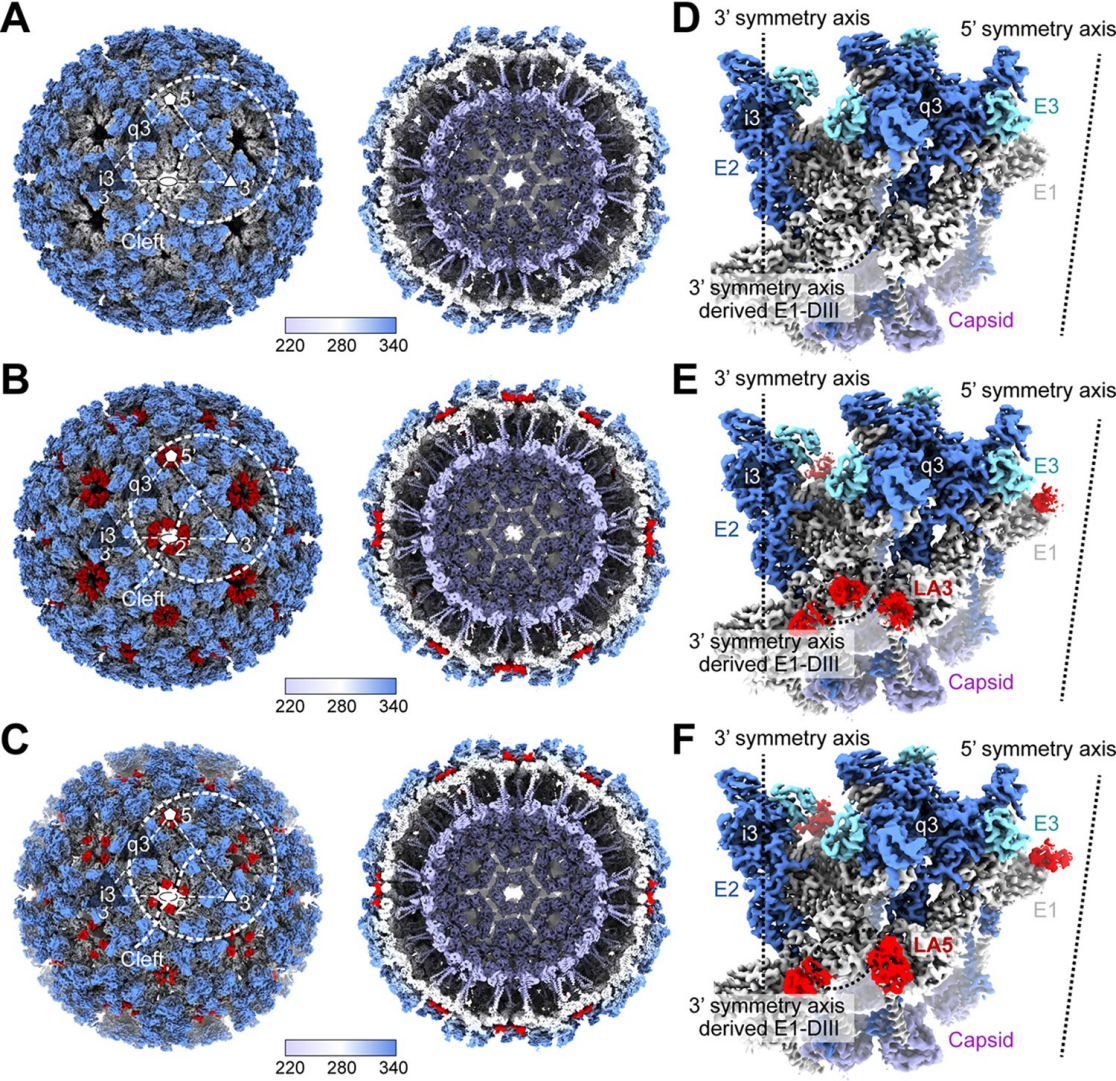
Structures of naked SFV virion, SFV in complex with LA3, and SFV in complex with LA5. (**A-C**) Left: Three-dimensional reconstructions of naked SFV virion (A), SFV in complex with LA3 (B), and SFV in complex with LA5 (C) viewed along the icosahedral twofold axis. All surfaces are colored radially from blue through white to purple from the lowest to highest radius. One icosahedral asymmetric unit is marked with a white triangle in all reconstructions. Icosahedral symmetry axes are drawn in white and labeled. The white dash line cycles are used to label the block region using in block-based reconstruction algorithm. Right: Thin slices of central sections of the naked SFV virion, SFV in complex with LA3, and SFV in complex with LA5 viewed along the twofold axis. The surfaces are colored the same as the left part. Both LA3 and LA5 are colored red. (**D-F**) Right: Surface-rendered representations showing cryo-EM density maps of asymmetric units zoned from naked SFV virion (D), SFV in complex with LA3 (E), and SFV in complex with LA5 (F). The E1, E2, capsid of SFV, and LA repeats (LA3 and LA5) are colored white, blue, purple, and red, respectively.

To elucidate the precise mechanism of SFV-VLDLR recognition, the single LA repeats (LA2-Fc, LA3-Fc, or LA5-Fc) in complex with the native SFV virion and SFV virion alone were firstly reconstructed by cryo-EM. Normal reconstruction strategies yielded an overall structure at a resolution of 8.8 Å (bin4) with icosahedral symmetry imposed. To increase the resolution, a block-based reconstruction algorithm [35,36] was applied, which improved the resolutions of SFV virion, SFV-LA3-Fc, and SFV-LA5-Fc to 3.02 Å, 3.45 Å, and 3.55 Å, respectively (S2 Fig). We failed in constructing the structure of the SFV-LA2-Fc complex, in which the density of LA2 could not be identified, likely due to its extremely low binding affinity for SFV. We assembled the asymmetric units into a completely symmetrical virus particle (Fig 2A to 2C). All the backbones as well as many side chains of the E1, E2, E3, capsid protein, LA3, and LA5 were clearly defined in the cryo-EM maps (S2 Fig). The asymmetric unit of the naked virus contains four copies of E1, E2, E3, and capsid protein (Fig 2D). In the structure of LA3-SFV, LA3 binds to the DIII domain of each E1 protein at both the 5’ and 2’ symmetry axis regions (Fig 2B and 2E). It is worth noting that LA5 was also observed to bind to E1-DIII at the 5’ and 2’ symmetry axis regions in the structure of LA5-SFV. However, in the twofold symmetry axis region, LA5 only binds to 2/3 of the E1-DIIIs (Fig 2C and 2F).

**Fig 3:**
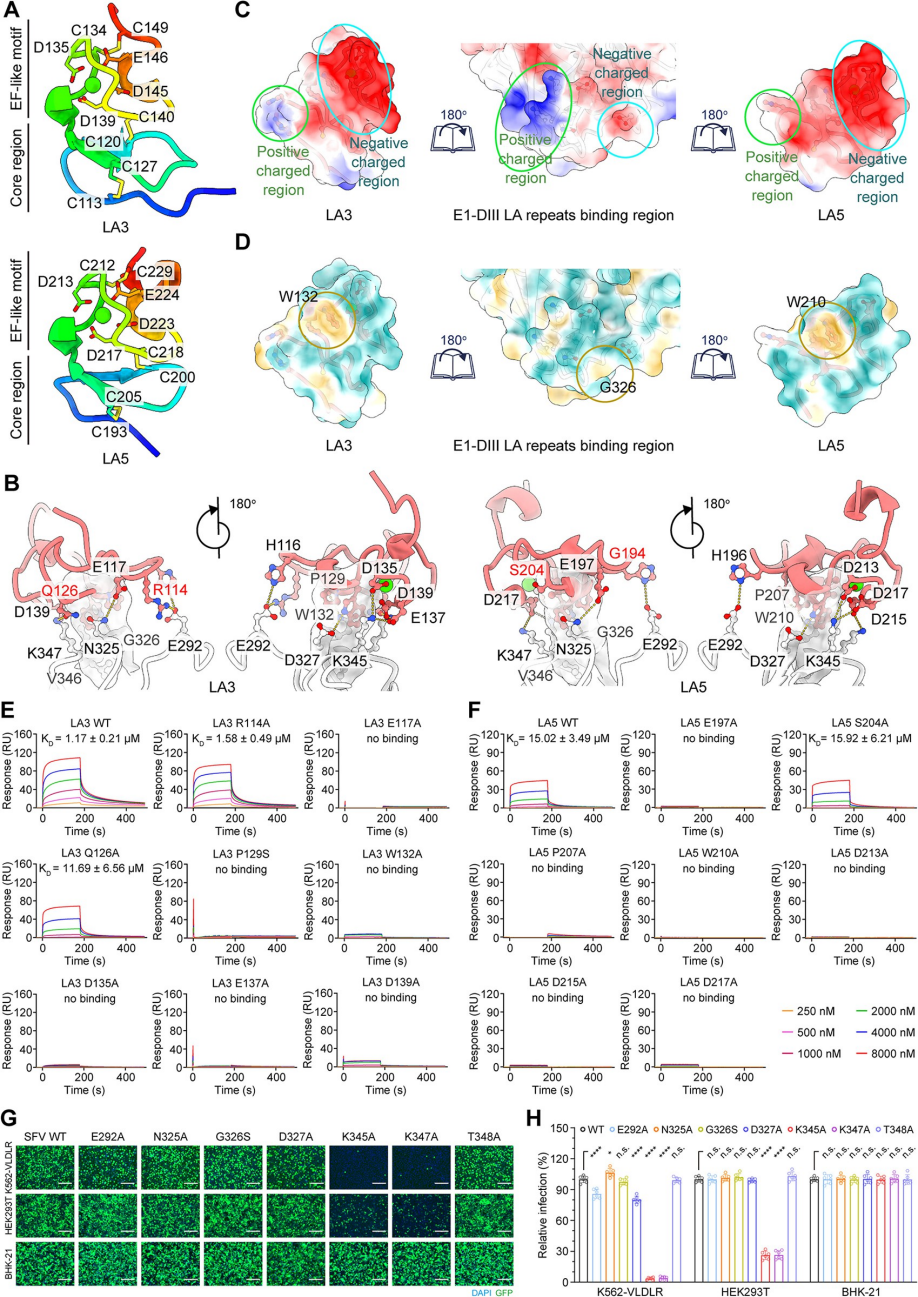
Detailed contacts between LA repeats and SFV virion. (**A**) Ribbon diagrams depicting the structure of LA3 (top) and LA5 (bottom), color-coded in a rainbow gradient from the N-terminus to the C-terminus. Calcium ions are represented as green spheres. All disulfide bonds and the ion-relative residues with negative charges are highlighted. (**B**) Ribbon-rendered representations showing details of the contacts between bound LA repeats (the left one is LA3 and the right is LA5) and SFV E1-DIII. Residues involved in key salt bridges, hydrogen bonds, and the formation of hydrophobic patches at the interface are shown as sticks. The differences between LA3 and LA5 are highlighted in red. (**C**) Surface-rendered representations showing the electrostatic potential maps of the binding interface of LA3 (left), LA5 (right), and E1-DIII (middle). Both positive (blue) and negative (red) charged regions involved in LA binding are highlighted. The color of the molecular surface ranges from blue (positive charged) through white to red (negative charged). (**D**) Surface-rendered representations showing the molecular lipophilicity potential maps of the binding interface of LA3 (left), LA5 (right), and E1-DIII (middle). The color on the molecular surface ranges from dark cyan (most hydrophilic) through white to dark goldenrod (most lipophilic). (**E and F**) A total of eight LA3 mutants (E) and seven LA5 mutants (F) were tested for their ability to bind to SFV by SPR. SFV was immobilized to 2,500 response units (RU) on a CM5 chip using standard amine coupling chemistry and was then tested for binding with gradient concentrations of LA3 or LA5 mutants. The data were analyzed with Biacore 8K evaluation software (GE Healthcare). K_D_ values are shown as mean ± SEM of three independent experiments. (**G**) Infection of K562 cells expressing full-length VLDLR, HEK293T cells, or BHK-21 cells with SFV-eGFP bearing E1 mutation at the receptor binding interface. Cells were imaged by fluorescence microscopy. Scale bar, 150 μm. The experiment was performed twice independently with similar results and representative images are shown. (**H**) Infection of K562-VLDLR cells, HEK293T cells, or BHK-21 cells with the SFV-eGFP mutants by FACS. Mean ± SD of three experiments (n = 6), One-way ANOVA with Tukey’s multiple comparisons test, ****P<0.0001; *P<0.05; n.s., not significant.

Both LA3 and LA5 share a similar overall architecture consisting of a core region at the N-terminus and an EF-like motif at the C-terminus. The disulfide bonds of LA3, which include C113-C127, C120-C140, and C134-C149, correspond to the counterparts C193-C205, C200-C218, and C212-C229 in LA5 (Fig 3A). Similarly, the residues in LA3 that chelate Ca2^+^ ions also have corresponding counterparts in LA5 (Fig 3A). What sets LA3 apart from LA5 is the length of the loop between the N-terminal strands which in LA3 is two residues longer than in LA5. This is a distinctive feature among all LA repeats of VLDLR (S3A and S3B Fig).

### The contact interface between LA domains and SFV

We further analyzed the binding details at the interface between the SFV and LA3 or LA5. We found that LA3 engages with the beta-sheet of E1-DIII through its EF-like motif (Fig 3B), creating an interaction area of 323 Å^2^. The residues R114, E117, Q126, D135, E137, D139, and H116 on LA3 form hydrogen bonds and salt bridges with E1’s K347, K345, N325, E292, and D327 (Fig 3B and 3C). Meanwhile, LA3’s P129 and W132 engage in weak hydrophobic interactions with E1’s G326 (Fig 3D). In the case of LA5, residues E197, D213, D215, D217, H196, P207, and W210, which correspond to the residues at the binding interface of LA3, sustain interactions with E1’s K347, K345, N325, E292, D327, and G326 (Fig 3B to 3D). However, the sites in LA5 that correspond to LA3’s Q126 and R114 are occupied by S204 and G194, whose side chains are too short to form hydrogen bonds with K347 and E292 of E1, resulting in a weaker binding affinity compared to LA3 (Fig 1B and 1C).

### Key interaction residues as confirmed by mutagenesis

To investigate the key residues involved in interactions between SFV and its receptor, we performed site-directed mutagenesis on LA3 and LA5 and used SPR to evaluate the binding affinity of these mutants to the SFV particles. As expected, most single mutations (E117A, P129S, W132A, D135A, E137A, and D139A) in LA3 resulted in a loss of SFV binding ability, while R114A and Q126A reduced the LA3 binding affinity to the virus (Fig 3E). Apart from the amino acid R114, these findings are consistent with the conclusion of Cao et al.[33]. Similar to the results with LA3, single mutations (E197A, P207A, W210A, D213A, D215A, and D217A) in LA5 led to a complete loss of SFV binding capacity, while S204A, as an exception, had no impact on the binding of LA5 to SFV due to its inability to form a salt bridge with E1’s K347 (Fig 3F).

We further mutated the residues at the interface of SFV E1-DIII, rescued the mutated virus, and assessed its impact on viral infection in BHK-21, HEK293T, and K562 cells that express the wild-type VLDLR (Fig 3G and 3H). The results showed that none of the seven substitutions (E292A, N325A, G326S, D327A, K345A, K347A, and T348A) in E1 affected the efficiency of SFV infection in BHK-21 cells. However, the mutants with E1 K345A or K347A substitutions exhibited a 70% decrease in infectivity to HEK293T. Notably, the infectivity of the K345A and K347A mutants was completely abolished in K562-VLDLR cells. This indicates that these two residues are the most critical amino acids for binding the virus to the receptor VLDLR. Additionally, the infectivity changes caused by G326S (slight decrease), D327A (slight decrease), and N325A (significant increase) in K562-VLDLR cells suggest the presence of hydrophobic interactions between P129 in LA3 (corresponding to P207 in LA5) and W132 (corresponding to W210 in LA5) and E1 (Fig 3G and 3H). Collectively, the findings from the mutagenesis assays affirm the key residues involved in the interactions between VLDLR and the virion.

### Binding patterns and molecular mechanism of individual LA binding with SFV

**Fig 4:**
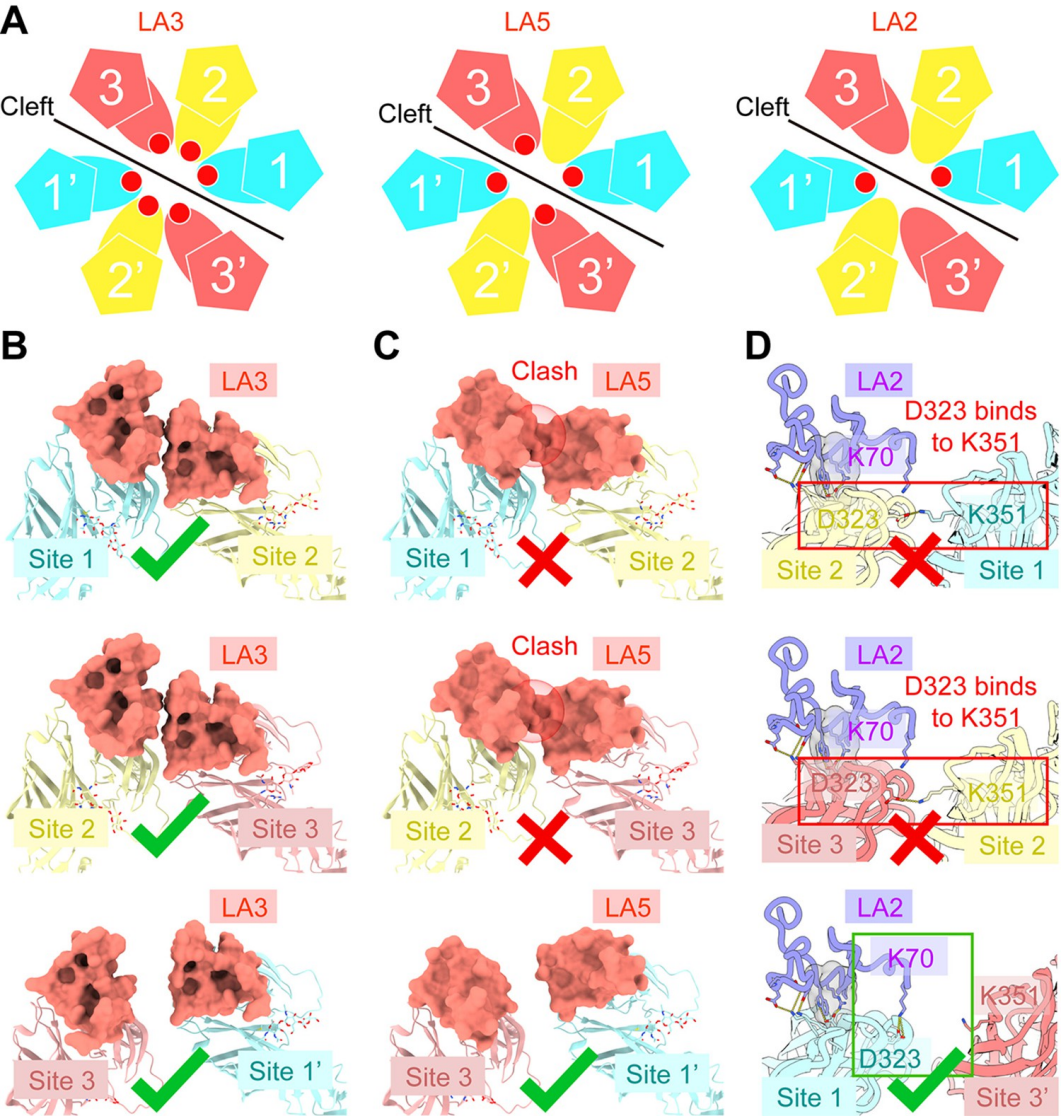
The binding pattern of LA repeats to SFV virion in a twofold axis region. (**A**) Three binding modes of LA repeats binding to SFV virion twofold axis region. LA3 binding to all the potential binding sites in the twofold axis region (left), LA5 binding to site-1, -3, -1’, and -3’ in the twofold axis region (middle), and LA2 binding to site-1 and site-1’ in the twofold axis region (right). All the bound LA repeats are shown as red points, and site-1 (or 1’), -2 (or 2’), and -3 (or 3’) are colored cyan, yellow, and hot pink, respectively. (**B**) Ribbon- and surface-rendered representations showing two LA3s simultaneously binding to adjacent LA repeats binding sites (top: LA3s binding to site-1 and -2; middle: LA3s binding to site-2 and -3; bottom: LA3s binding to site-3 and -1’). LA3s are shown as surface and colored coral red. Site-1 (or 1’), -2, and -3 of SFV twofold axis region are shown in ribbon and colored cyan, yellow and hot pink, respectively. (**C**) Ribbon- and surface-rendered representations showing the fitting models of two LA5s simultaneously binding to adjacent LA repeats binding sites (top: LA5s binding to site-1 and -2; middle: LA5s binding to site-2 and -3; bottom: LA5s binding to site-3 and -1’). LA5s are shown as surface and colored coral red. Site-1 (or 1’), -2, and -3 of SFV twofold axis region are shown as ribbon and colored cyan, yellow, and hot pink, respectively. The clashes between site-1 bound LA5 or site-3 bound LA5, and site-2 bound LA5 are highlighted. (**D**) Ribbon-rendered representations showing simulation of two LA2s simultaneously binding to adjacent LA repeats binding sites (top: LA2s binding to site-1 and -2; middle: LA2s binding to site-2 and -3; bottom: LA2s binding to site-3 and -1’). Site-1, -2, -3 (or 3’) of SFV twofold axis region and AlphaFold2 predicted structure of LA2 are colored cyan, yellow, hot pink, and purple, respectively. Residues involved in conformational change are labeled and shown as sticks.

The sequences of LA repeats in VLDLR exhibit over 25% identity, and superimpositions of their structures, generated by AlphaFold, indicate a high structural similarity with RMSDs ranging from 0.4~0.9 Å (S3A and S3B Fig, and). However, different LA repeats exhibit distinct binding modes with SFV in the fivefold and twofold axis regions (Fig 2B and 2C). In the fivefold axis region, LA3 and LA5 exhibit a spatially hindered, unsaturated distribution (S3C Fig) and LA2 doesn’t bind in this region, according to the structure of LA1-2-SFV [33]. All three LAs exhibit distinct binding modes with SFV in the twofold axis region. We designate the binding site to clarify these different binding modes as depicted in Fig 4A. In this arrangement, LA3 binds to all positions within site-1-3 and site -1’-3’, LA5 binds to site-1, -3, -1’, and -3’, while LA2 only binds to site-1 and site-1’ (Figs 2B, 2C and 4A).

To gain insights into the molecular mechanisms underlying the diverse binding modes of LA, it was initially necessary to ascertain whether receptor binding resulted in alterations to the conformation of the virus. A comparison between the receptor-bound and exposed twofold axis regions of the virus was made through superimposition, revealing no significant conformational changes (S3D Fig). Further comparisons of receptor-binding sites within sites 1 to 3 (or sites 1’ to 3’) demonstrated that all binding sites of E1-DIII in the twofold symmetry axis region possess identical interfaces with the LA repeats (S3E Fig). Thus, receptor engagement neither induces conformational changes in the virion nor alters the binding patterns of LA repeats.

To further elucidate the molecular mechanisms underlying the binding patterns of individual LA repeats to SFV, we employed the AlphaFold2 software to predict the structure of LA2 in a complex with E1-DIII. Subsequently, we built the structures of LA2, LA3, and LA5 binding at all potential binding sites in the twofold axis region through superimposition. Analysis of the fully saturated LA-bound structures revealed that LA3 can bind independently at site-1-3 and site-1’-3’, satisfying the conditions for simultaneous binding at all six sites (Fig 4A and 4B). LA5 can also independently bind to site-1 to -3 and site-1’ to -3’, but when LA5 binds to site-2 (or 2’), it creates steric hindrance with the LA5 binding at site-1 (or 1’) and site-3 (or 3’). Therefore, in the presence of an excess of LA5, LA5 tends to bind at site-1 (or 1’) and site-3 (or 3’) to avoid steric clashes and maximize occupancy at available binding sites (Fig 4A and 4C). In the binding mode of LA2, D323 of E1-DIII at site-1 (or site-1’) forms a salt bridge with K70 from LA2. However, at site-2 (or 2’) and -3 (or 3’), the absence of cleft leads to the formation of the salt bridge between D323 in this binding site and K351 from the adjacent binding site, thereby preventing interaction between K70 from LA2 and the viral LA binding site (Fig 4A and 4D). This binding mode also elucidates why LA1-2 cannot bind to the fivefold symmetry axis region as LA3 or LA5. In the fivefold axis region, all D323s of E1-DIIIs are occupied by K351 resulting in the abolishment of the binding of LA2.

### LA2, LA3, and LA5 domains synergistically promote attachment and entry of SFV

**Fig 5:**
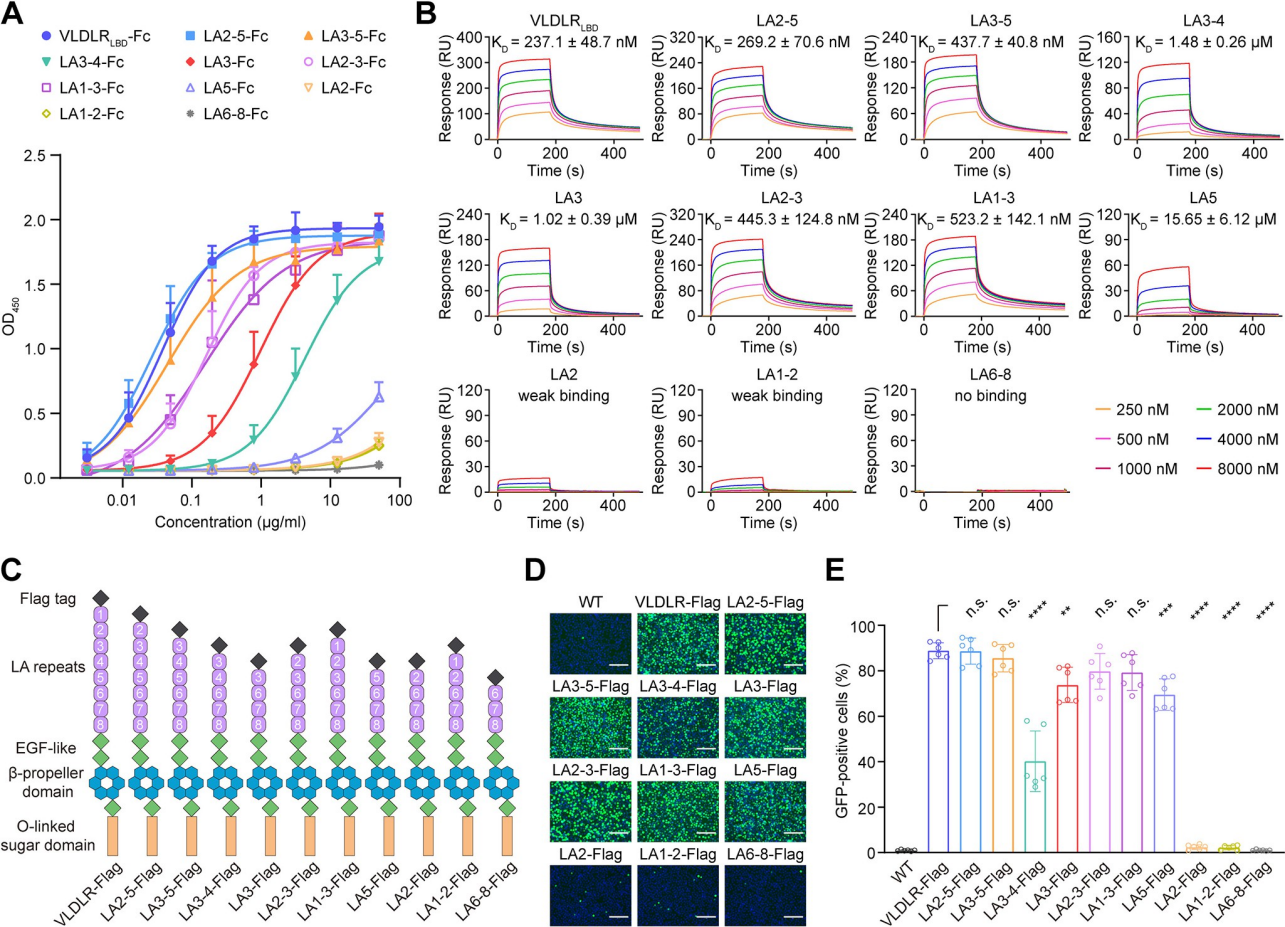
LA2, LA3, and LA5 synergistically promote cell attachment and entry of SFV. (**A**) Binding of individual LA2, LA3, LA5, and LA concatemers to SFV virions measured by ELISA. Mean ± SD of three experiments (n = 6). OD_450_, optical density at 450 nm. (**B**) Binding affinity of individual LA2, LA3, LA5, and LA concatemers to SFV virions measured by SPR. K_D_ values are shown as mean ± SEM of three independent experiments. (**C**) VLDLR ectodomain and deletion constructs. LBD LA repeats are numbered. (**D**) Infection of wild-type K562 cells or K562 cells expressing indicated constructs shown in (C) with SFV-eGFP. Cells were imaged by fluorescence microscopy: scale bar, 150 μm. The experiment was performed twice independently with similar results and representative images are shown. (**E**) Infection of wild-type or transduced K562 cells with SFV-eGFP measured by FACS. Mean ± SD of three experiments (n = 6), One-way ANOVA with Tukey’s multiple comparisons test, ****P < 0.0001; ***P < 0.001; **P < 0.01; n.s., not significant.

Considering that the LA repeats exist as concatemer in VLDLR, it is essential to delineate the synergistic effects among these repeats to construct an accurate VLDLR-mediated SFV entry model. To further analyze whether the LA repeats synergistically bind SFV, we split LA repeats into concatemers and produced LA2-5, LA3-5, LA3-4, LA2-3, LA1-3, LA1-2, and LA6-8. VLDLR and LA2-5 exhibited the highest affinity in the ELISA-based binding assay and SPR analysis (with K_D_ of 252.3 nM and 269.2 nM, respectively) (Fig 5A and 5B). LA3-5 and LA2-3 exhibited higher affinities, with K_D_ values of 437.7 nM and 445.3 nM respectively, compared to LA3, which had a K_D_ of 903.0 nM. This suggests that LA2, LA3, and LA5 may create a synergistic effect in the binding of VLDLR to SFV. Compared to LA2-3, LA1-3 exhibited a slight decrease in affinity, while LA1-2 exhibited a binding affinity similar to LA2, indicating that LA1 does not interact with the virus (Fig 5A and 5B). Compared with LA3, LA3-4 showed weaker binding to SFV (with a K_D_ of 1.48 μM), indicating that LA4 does not participate in virion binding. LA6-8 did not bind to SFV as indicated by the SPR results. Furthermore, all the results of SPR were confirmed by receptor-mediated viral entry assays (Fig 5C to 5E). Taken together, these results show that VLDLR LA3, LA5, and LA2 work synergistically to promote the attachment and entry of SFV into host cells.

### Molecular basis of the synergistic effects of LAs in concatemers binding to SFV

**Fig 6:**
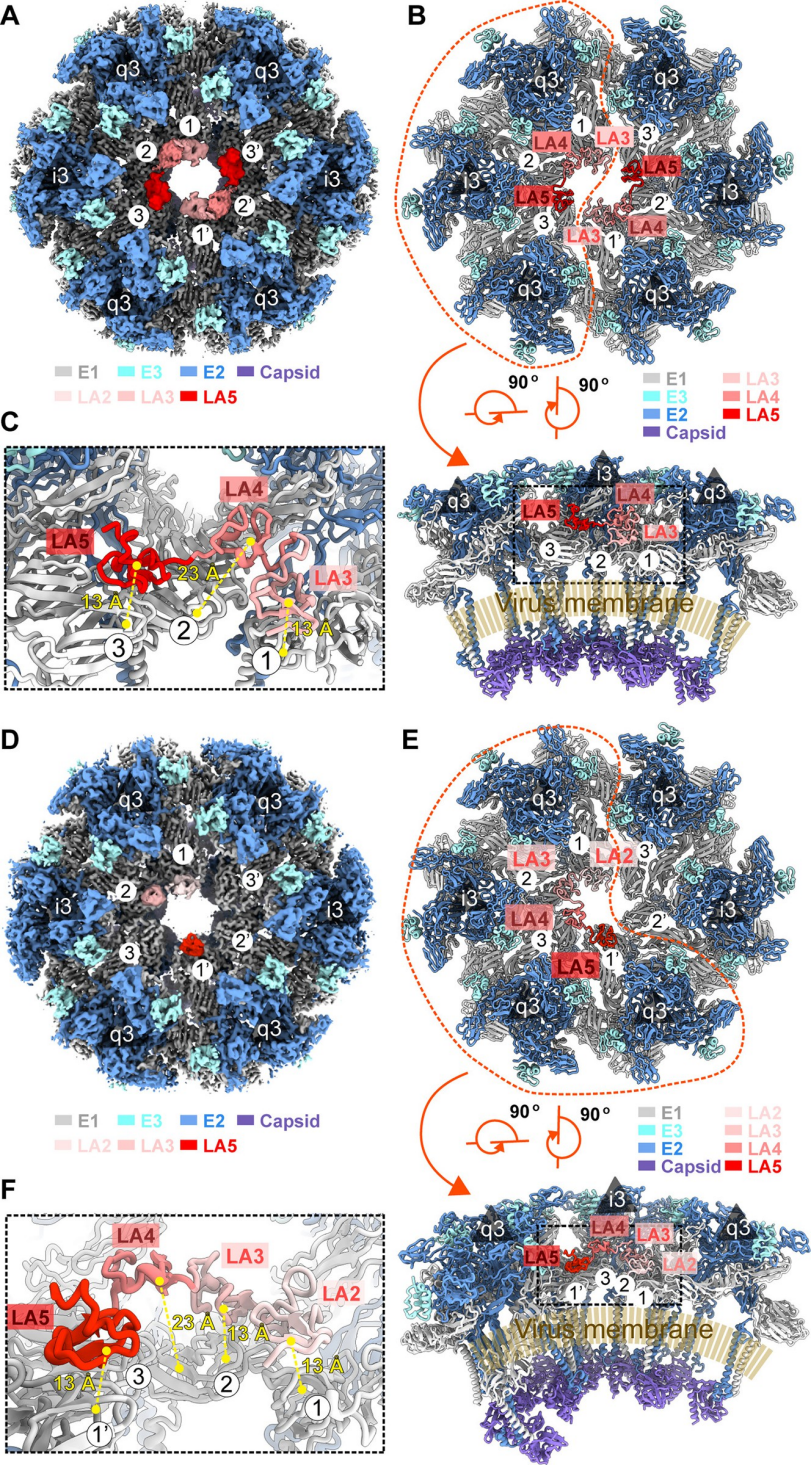
The structures of LA3-5 and VLDLR LBD in complex with SFV virion. (**A**) Surface-rendered representations showing cryo-EM density maps of twofold axis region from SFV virion in complex with LA3-5 concatemer. The E1, E2, capsid of SFV, and LA repeats (LA3-5) are colored white, blue, purple, and red, respectively. All the LA repeats binding sites, the icosahedral threefold axis (i3), and the quasi-threefold symmetry axis (q3) are labeled. (**B**) Top: Ribbon-rendered representation showing the model of receptor binding sites in LA3-5 concatemer bound twofold axis region. All the LA repeats binding sites, the icosahedral threefold axis (i3), and the quasi-threefold symmetry axis (q3) are labeled. Bottom: Sideview of ribbon-rendered representation of the area delineated by the red dashed cycle in the top view. The LA repeats binding sites, the icosahedral threefold axis (i3), and the quasi-threefold symmetry axis (q3) are labeled. The membrane inside the SFV virion is shown as a brown fence. (**C**) Zoom-in view of the ribbon-rendered representation of the area boxed with a black dash box shown at the bottom in panel B. Yellow labels show distances between centers of LAs and their binding sites. The distance between the bound LA and the binding site is measured as 13 Å. (**D**) Surface-rendered representations showing cryo-EM density maps of twofold axis region derived from SFV virion in complex with low-dose VLDLR LBD. The E1, E2, capsid of SFV and LA repeats are colored white, blue, purple, and red, respectively. All the LA repeats binding sites, the threefold axis (i3), and the quasi-symmetry axis (q3) are labeled. (**E**) Top: Ribbon-rendered representation showing the model of VLDLR (LA2-5) bound twofold axis region receptor binding sites. All the LA repeats binding sites, the icosahedral threefold axis (i3), and the quasi-threefold symmetry axis (q3) are labeled. (**F**) Zoom-in view of the ribbon-rendered representation of the area boxed with a black dash box shown at the bottom in panel E, Yellow labels show distances between centers of LAs and their binding site, showing the LA2-5 repeats simultaneously binding to different receptor sites of one twofold axis region. The distance between the bound LA and the binding site is measured as 13 Å.

Considering that VLDLR-SFV structures were generated under conditions of an excess of LAs, the high affinity of LA3 results in almost all receptor binding sites being occupied by LA3 [33]. However, the amount of VLDLR is much lower *in vivo*, leading to binding modes that might differ from the resolved states. To verify this hypothesis, we prepared the complex LA3-5-SFV at an unsaturated ratio of LA3-5 per virion, instead of excess concatemers. The structure was resolved to 8.8 Å (bin4) using icosahedral symmetry. Furthermore, a block-based reconstruction algorithm was utilized to optimize the resolutions of 2’ symmetry axis region units to 3.41 Å. At this resolution, both the receptor distribution and binding interfaces can be identified (S4 Fig). A comparison of the previously documented mode [33] using VLP and an excess of LA3-5 with the current binding mode revealed significant differences. These include the elimination of bound LA in the fivefold axis region and a smear density counterclockwise to the LA-bound density at site-1, which was observed and confirmed with a 15 Å low-pass filtered map (Fig 6A). This density was located at the C-terminus of the LA bound at site-1 and could be attributed to LA3’s C-terminal connected LA4. Importantly, the AlphaFold2 predicted structure of LA4 could match this relatively weak density, and the C-terminus of LA3-4, assembled based on the density, could connect with the N-terminus of LA5 bound at site-3 (Fig 6B). In this binding model, LA4 was situated between LA3 and LA5 and has no interaction with E1-DIII of site-2 (Fig 6C). However, due to the space limitation imposed by the binding of LA4, LA3, and LA5 can’t attach to neighboring two E1-DIIIs positioned counterclockwise. In addition, when LA3 binds to site-2, the LA5 from the same LA3-5 concatemer will bind to site-1’, which could create spatial hindrance for the adjacent binding of LA3 or LA5 at site-2 (Fig 4C). Therefore, the binding of unsaturated LA3-5 to the same side at site-1 and -3 may represent an *in vivo* attaching mode for SFV. The result also suggests that the in vivo binding mode of VLDLR to SFV may differ from previously observed patterns.

Inspired by the structure of the *in vivo* mimic structure of LA-3-5 in complex with SFV, VLDLR LBD-SFV was also prepared at a ratio of 30:1. This structure was resolved to 8.8 Å (bin4) using icosahedral symmetry, and the twofold axis region was optimized with the same algorithm (S4 Fig). After reconstruction of the particles at the twofold axis region, to avoid bias introduced by icosahedral symmetry, two rounds of 3D classifications were used to release the symmetry, and the asymmetric twofold axis region reconstruction at 3.7 Å resolution was produced (Figs 6D and S4). Distinct from LA3-5-SFV, VLDLR LBD-SFV displayed an asymmetric binding pattern, indicating a *bona fide in vivo* mimicry attachment. In the structure of VLDLR LBD-SFV, LA2, LA3, and LA5 bind to site-1, site-2, and site-1’, respectively, with a maximal interaction area between VLDLR LBD and SFV, facilitating viral attachment to host cells (Fig 6E and 6F). In addition, the properties of single LA binding modes, including LA2 only binding to site-1 (or site-1’), and binding of LA5 in site-2 (or site-2’) needs adjacent binding sites to stay empty, were also confirmed in this binding mode. In the fivefold axis region, LAs still exhibit a spatially hindered unsaturated distribution. The densities of the LAs bound to fivefold axis regions are significantly weaker compared to those in the twofold axis regions in both the *in vivo* LA3-5 and VLDLR binding modes, suggesting that receptor binding in the fivefold axis region is less functional.

### The conformational transition raised by multivalent binding facilitates SFV entry

**Fig 7:**
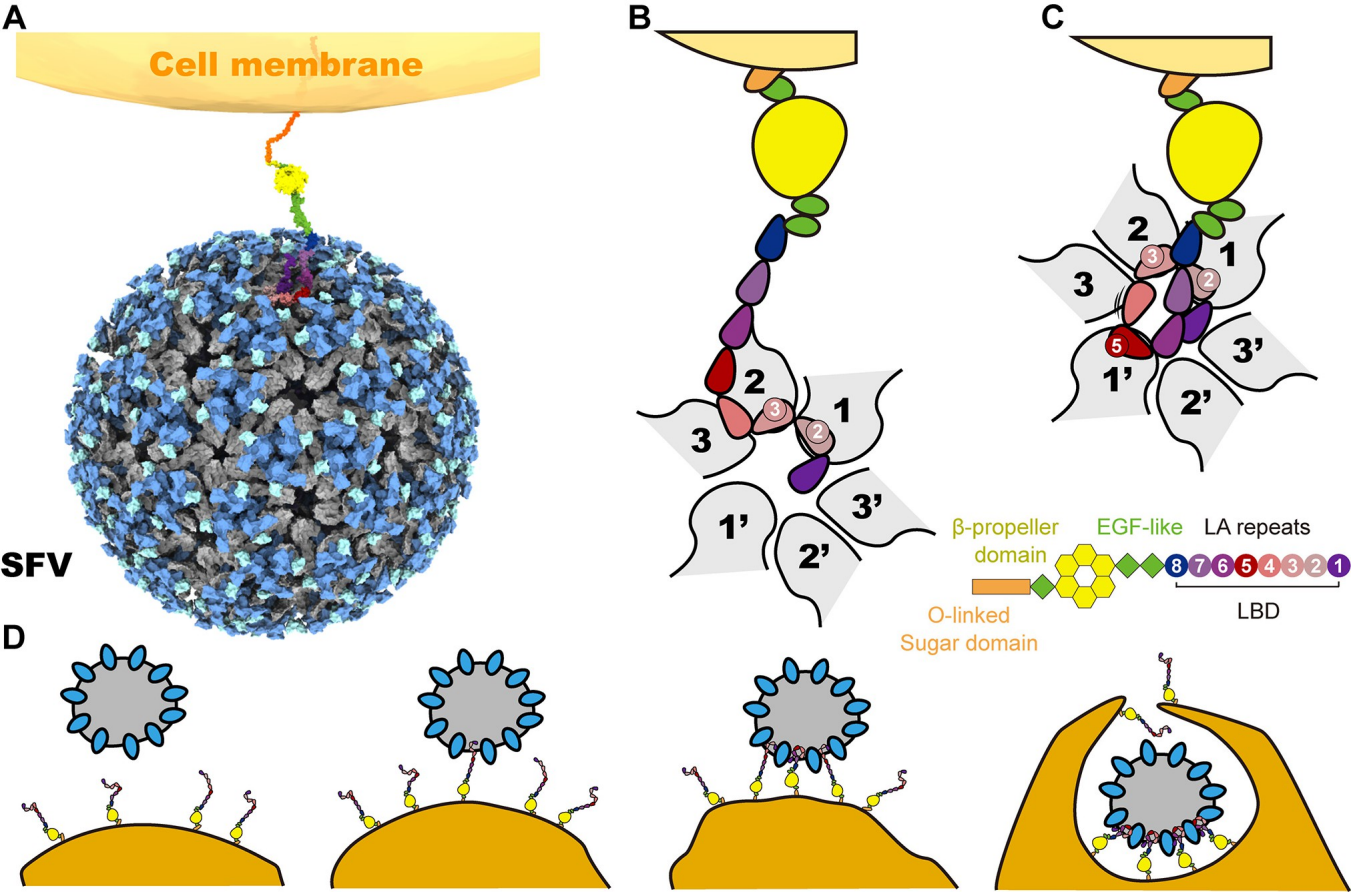
The proposed multivalent binding modes accelerated the endocytosis pathway for SFV. The surface-rendered representation shows SFV utilizes a twofold axis region binding to VLDLR to recognize host cell (**A**). The binding of VLDLR to the SFV virion occurs in two distinct states within the twofold axis region. Initially, the SFV virion engages LA2 and LA3 concurrently at receptor binding sites 1 and 2 (**B**, state 1). This action triggers the subsequent attachment of LA5 to receptor binding site-1’ (**C**, state 2). The binding of LA5 leads to a decrease in the distance between the SFV virion and the host cell, thereby facilitating the attachment of other VLDLRs present on the cell membrane surface to the SFV virion, which tiggers the formation of an endosome and promotes the entry of the SFV virion (**D**).

VLDLR is a member of the low-density lipoprotein receptor family, known for its role in regulating cellular lipid metabolism, particularly in response to very low-density lipoprotein [37]. Based on the various binding modes of individual LA domains and concatemers with SFV, coupled with previous observations on SFV’s cell entry [38], a model where multiple binding sites assist in the attachment of the virus can be hypothesized. Initially, VLDLR recognizes the closest E1-DIII of SFV through its cell distal LA2-3. At this stage, LA2 and LA3 each bind to site-1 and site-2 of SFV’s twofold axis region. Then, VLDLR’s LA5 binds to the site-1’ position on the same twofold axis region, bringing SFV closer to the cell’s surface. This enables the other LA2-3s of freely available VLDLRs, which would be unable to bind to SFV initially, to bind to vacant twofold or fivefold axis regions of SFV. Simultaneously, a conformational transition occurs from the binding modes of LA2-3 to LA2-5 in the twofold axis region, further reducing the distance between the cell membrane and SFV. Consequently, with the involvement of clathrin, an invaginated vesicle is formed, marking the completion of the virus’s entry initiation stage into the host cell (Fig 7A to 7D).

## Discussion

LA repeats around LA3 have been shown to promote the synergistic binding of multiple LAs to SFV, facilitating the entry of the virus into host cells [33]. However, details of the molecular mechanisms of how LAs around LA3 assist in this binding remain unclear. In this study, we can classify the modes of synergistic binding of LAs around LA3 to SFV and their impact on virus-receptor interaction into three types—1) The type 1 mode promotes a decrease in K_D_ from 900 nM to 500 nM. This enhancement is brought about by two interactions: (i) LA2-3 binding to SFV with LA2 binding to site 1 and LA3 binding to site 2 on the surface of the virus and (ii) LA3-5 binding to SFV with LA3 binding to site 1 and LA5 binding to site 3 on the surface of the virus. Both interactions involve an additional LA that increases the surface contact area and contributes to the stabilization of the binding interface when compared to the scenario of only one type, LA3, binding to SFV. 2) The type 2 mode leads to a decrease in K_D_ from 900 nM to 250 nM, which is brought about by the binding of either LA2-5 or VLDLR to SFV. In both interactions, LA2 binds to site 1, LA3 binds to site 2, and LA5 binds to site 1’. This configuration probably allows both LA2 and LA5 within one concatemer to stabilize LA3’s binding to SFV. 3) The type 3 mode was identified while comparing the binding affinities of LA concatemers for SFV. LA repeats without any noticeable binding affinity can reduce the overall affinity of binding units. For instance, LA4 in LA3-4 results in a 500 nM increase in K_D_ compared to LA3, and LA1-3 exhibits a 100 nM increase in K_D_ compared to LA2-3. LA1 and LA4 do not bind SFV (Fig 5B). Thus, the results of our binding assays coupled with cryo-EM data help dissect the contribution of LAs to the crucial step of entry of SFV into the host cell for establishing a successful infection. Importantly, they shed light on the intricate mechanisms by which the consecutive LA repeats around LA3 promote synergistic binding of LAs to SFV, leading to the internalization of the virus.

The SFV virion, instead of the recombinant protein E1-DIII, was used in an ELISA-based binding assay, entry-blocking assay, and SPR binding assay to mimic the *in vivo* conditions of a natural SFV infection (Figs 1 and 5). All these experiments revealed that LA3, LA5, LA2, not LA1 can bind to the virus and LA5 has a higher affinity than LA2, which conflicts with the results of a previous study performed using recombinant protein E1-DIII [33]. Recent studies have suggested that LA6 can bind to the spike protein of SFV [31, 34]. However, our study failed to demonstrate any binding between LA6-Fc or LA6-8-Fc proteins and SFV virions (Figs 1B, 1C, 5A, and 5B). Furthermore, LA6-Fc did not prevent SFV infection in HEK293T cells (Fig 1D and 1E). Additionally, overexpression of the LA6-8-Flag construct in the non-susceptible cell line K562 did not result in SFV infection (Fig 5D and 5E). Our findings align with those reported by Cao et al. [33]. This apparent discrepancy might be attribute differences in experimental materials and design across laboratories, or it could suggest an unknown mechanism of LA6 in mediating SFV cell entry. Further exploration of this topic is warranted in future studies.

Receptors with multivalent binding properties are commonly found in nature. Notably, receptors like VLDLR, LDLR, ApoER2, and LRP1 all possess multiple ligand binding sites that exhibit high structural similarity, and many viruses exploit these multi-ligand binding domains as their receptors [29,39,40]. However, different viruses may engage these types of receptors in various ways. For instance, SFV and EEEV, despite having similar spike glycoproteins, adopt different binding modes when interacting with VLDLR [30,33]. This suggests that these two alphaviruses may have evolved distinct mechanisms for using VLDLR as their cell entry receptor. They engage host receptors through clusters of relatively conserved acidic and aromatic residues. Despite having only a small interface at each site, they bind at multiple locations, resulting in high-avidity interactions. This binding strategy is remarkably similar to that of physiological ligands for LDLR-related proteins. Such diversity complicates the development of broad-spectrum anti-alphavirus drugs that target virus-receptor interactions.

In this study, the structures we analyzed, including LA3-SFV, LA5-SFV, and the previously reported LA1-2-SFV VLP, validate the distinct binding modes of LA with the virus and reveal the potential binding modes *in vivo*. The structural analysis of the LA3-5-SFV complex at a lower stoichiometric ratio, along with the structure of the VLDLR-SFV complex confirms the LA binding models and unveils multiple states of VLDLR binding to the virus during the natural invasion of the host cells by the virus. Our findings provide solid evidence for the multi-LAs-binding-induced conformational effects that mediate the cellular internalization of large cargo, which have important implications for the development of antiviral drugs.

## Materials and methods

### Cells and viruses

HEK 293T (human kidney epithelial, ATCC CRL-11268) and BHK-21 (baby hamster kidney, ATCC CCL-10) cells were maintained in Dulbecco’s modified Eagle’s medium (DMEM, Gibco) supplemented with 10% (v/v) fetal bovine serum (FBS) and 1% (v/v) penicillin-streptomycin (Thermo Fisher Scientific). K562 (human chronic myelogenous leukemia, ATCC CCL-243) cells were maintained in Iscove’s modified Dulbecco’s medium (IMDM, Gibco) supplemented with 10% FBS and 1% penicillin-streptomycin. SFV-eGFP is a recombinant SFV that was derived from a full-length cDNA clone of the SINV strain YN_222 genome (MH229928) in which the coding region of the structural proteins was replaced by that of SFV (SFV4, KP699763) and inserted an enhanced-green fluorescent protein (eGFP) gene and an additional sub-genomic (SG) promoter between the original SG promoter and structural genes. The virus was propagated in BHK-21 cells and was titrated by standard plaque assays.

### Recombinant VLDLR protein generation and purification

Gene fragments (VLDLR_LBD_, residues 31–355; LA1, residues 31–70; LA2, residues 69–111; LA3, residues 110–152; LA4, residues 151–191; LA5, residues 190–237; LA6, residues 236–276; LA7, residues 275–316; and LA8, residues 315–355; LA2-5, residues 69–237; LA3-5, residues 110–237; LA3-4, residues 110–191; LA2-3, residues 69–152; LA1-3, residues 31–152; LA1-2, residues 31–111; LA6-8, residues 236–355) encoding the VLDLR protein (NP_003374.3) were codon-optimized, synthesized (GenScript) and inserted into the pCDNA3.4 vector with the native signal peptide sequence, followed by an HRV 3C cleavage site (LEVLFQGP) and the mouse IgG2b Fc region. The RAP chaperone protein (residues 1–357; GenBank: NM_002337) was cloned into the pCDNA3.4 vector. HEK 293F cells (1 L) were seeded at 1.5 × 10^6^ cells per ml and then transfected with 1 mg of these VLDLR Fc plasmids and 0.5 mg of RAP in diluted Opti-MEM complexed with polyethyleneimine (PEI) transfection reagent (Sigma). The supernatant was collected 72 h after transfection, centrifuged at 5,000 g for 10 min, and purified using Protein A Resin FF (GenScript, L00464). VLDLR Fc fusion proteins were separated from RAP by binding the complex to protein A resin and washing with 100 column volumes of 10 mM EDTA in TBS, followed by a wash with 50 column volumes of 10 mM EDTA and 500 mM NaCl in TBS. Then, the Fc fusion proteins were refolded on the column by washing with 100 column volumes of TBS containing 2 mM CaCl_2_ and eluted using the manufacturer’s protocol. The proteins were repurified by size-exclusion chromatography using Superdex 200 Increase 10/300GL (Cytiva). The VLDLR Fc fusion proteins were stored in TBS containing 2 mM CaCl_2_. Protein purity was confirmed by SDS–PAGE analysis. The mutant LA3-Fc and LA5-Fc plasmids were constructed by site-directed mutagenesis with each of the identified residues involved in binding SFV separately.

### Virus production and purification

SFV virions were purified as described previously [18]. In brief, BHK-21 cells were grown to 95% confluence and infected with SFV at a multiplicity of infection (MOI) of 0.1. The supernatant was harvested 24 hours post-infection and centrifuged to remove cell debris. The virions were precipitated with a 20% (w/v) sucrose cushion at 100,000 × g for 1.5 h at 4°C. The pellet was gently resuspended in phosphate-buffered saline (PBS, pH 7.4) and the virions were purified using ultracentrifugation by loading the resuspended virus onto a 20–45% (w/v) sucrose discontinuous density gradient and spinning it at 100,000 × g for 2 h at 4°C. The virus band was collected from the gradient using a syringe. The sucrose was removed through buffer exchange with Tris-buffered saline (TBS) at pH 7.4 (for Cryo-EM sample preparation) or in PBS (for SPR experiments). The virions were concentrated using an Amicon Ultra-4 100-kDa cut-off filter (Sigma). The purity and integrity of the viral particles were examined by negative-staining electron microscopy.

### ELISA-based VLDLR-Fc binding assays

The purified SFV were immobilized (1.5 μl, 2 mg/ml) onto ELISA plates (Corning Costar) overnight in 50 mM carbonate buffer (35 mM NaHCO_3_, 15 mM Na_2_CO_3_), pH 9.6. Plates were washed with PBST buffer (0.05% Tween-20 in PBS) and then blocked with PBS supplemented with 4% BSA (blocking buffer) for 1 h at room temperature. Subsequent washing was followed by incubation with indicated concentrations of VLDLR_LBD_-Fc, LA domain of VLDLR Fc fusion, and negative MXRA8_ect_-Fc control diluted in 2% BSA (binding buffer) for 1 h at room temperature. After serial washes with PBST, plates were incubated with horseradish peroxide conjugated goat anti-mouse IgG (H + L) at 1:3000 dilution in binding buffer (Sigma, 71045-M) for 1 h at room temperature. After washing with PBST, plates were incubated with 3,3′ - 5,5′ tetramethylbenzidine substrate (Solarbio), stopped with 2 M H_2_SO_4_ and optical density was assayed by an ELx808 Microplate Reader (BioTek) at 450 nm. All data presented are representatives of at least three independent experiments.

### Surface plasmon resonance (SPR) analysis

The binding kinetics and affinity of VLDLR to SFV were analyzed by SPR using a Biacore 8K system (GE Healthcare) in PBS buffer containing 0.05% Tween-20 (PBST, pH 7.4) at 25°C. The purified SFV virions were immobilized on a CM5 sensor chip (GE Healthcare, BR100530) into different channels with more than 2,500 response units through PBST. Then, VLDLR_LBD_-Fc, LA1-Fc, LA2-Fc, LA3-Fc, LA4-Fc, LA5-Fc, LA6-Fc, LA7-Fc, LA8-Fc, LA2-5-Fc, LA3-5-Fc, LA3-4-Fc, LA2-3-Fc, LA1-3-Fc, LA1-2-Fc, LA6-8-Fc, or LA3-Fc, LA5-Fc mutant fusion proteins were diluted with PBST, and the gradient concentrations of proteins were passed over the chip surface at a rate of 30 μL/min. After each injection cycle, a solution containing 1M NaCl was used to regenerate the chip. The binding kinetics were analyzed and affinity constants were calculated using Biacore Insight Evaluation software (GE Healthcare).

### Entry-blocking assays with VLDLR-Fc fusion proteins

For blocking studies with SFV-eGFP chimeras, we pre-mixed chimeras at an MOI of 5 with serially diluted VLDLR_LBD_-Fc, LA1-Fc, LA2-Fc, LA3-Fc, LA4-Fc, LA5-Fc, LA6-Fc, LA7-Fc, LA8-Fc, or MXRA8_ect_-Fc fusion proteins for 30 min at 37°C, then added mixtures to HEK 293T cells and incubated for 1 h at 37°C, and then replaced the media. We visualized cells by fluorescence microscopy using an EVOS M5000 microscope (Thermo Fisher Scientific) or measured GFP expression by FACS using a Cytomics TM FC 500 (Beckman Coulter) at 12 h post-infection for SFV-eGFP chimeras, after washing cells twice with PBS and then fixing them with PBS containing 2% (v/v) formalin. All flow cytometry data were processed using FlowJo (FlowJo). We calculated relative infection as follows: Relative infection (%)  =  (%GFP-positive cells in the presence of the Fc fusion protein) / (%GFP-positive cells in the absence of Fc fusion) × 100%.

### Ectopic expression experiments

To construct VLDLR truncation mutants containing an N-terminal Flag tag, we introduced the sequence encoding a glutamic acid residue followed by a Flag tag (DYKDDDDK) and a short linker (GSG) at the downstream of the signal sequence to monitor cell surface expression. All constructs were codon-optimized, synthesized, and inserted into the Lentivirus vector pLVX-IRES-Puro (Takara Clontech). The following VLDLR constructs were generated: full-length (VLDLR-Flag, residues 1–873), LA1 linked to LA6 + LA7 + LA8 + EGF-like domains + β-propeller domains + an O-linked sugar domain + transmembrane (TM) + cytoplasmic domains (CYT) (LA1-Flag, residues 31–70 and 236–873), LA2 linked to LA6 + LA7 + LA8 + EGF-like domains + β-propeller domains + an O-linked sugar domain + + TM + CYT (LA2-Flag, residues 69–111 and 236–873), LA3 linked to LA6 + LA7 + LA8 + EGF-like domains + β-propeller domains + an O-linked sugar domain + TM + CYT (LA3-Flag, residues 110–152 and 236–873), LA5 linked to LA6 + LA7 + LA8 + EGF-like domains + β-propeller domains + an O-linked sugar domain + TM + CYT (LA5-Flag, residues 190–873), LA2 + LA3 + LA4 + LA5 linked to LA6 + LA7 + LA8 + EGF-like domains + β-propeller domains + an O-linked sugar domain + TM + CYT (LA2-5-Flag, residues 69–873), LA3 + LA4 + LA5 linked to LA6 + LA7 + LA8 + EGF-like domains + β-propeller domains + an O-linked sugar domain + TM + CYT (LA3-5-Flag, residues 110–873), LA3 + LA4 linked to LA6 + LA7 + LA8 + EGF-like domains + β-propeller domains + an O-linked sugar domain + TM + CYT (LA3-4-Flag, residues 110–191 and 236–873), LA2 + LA3 linked to LA6 + LA7 + LA8 + EGF-like domains + β-propeller domains + an O-linked sugar domain + TM + CYT (LA2-3-Flag, residues 69–152 and 236–873), LA1 + LA2 + LA3 linked to LA6 + LA7 + LA8 + EGF-like domains + β-propeller domains + an O-linked sugar domain + TM + CYT (LA1-3-Flag, residues 31–152 and 236–873), LA1 + LA2 linked to LA6 + LA7 + LA8 + EGF-like domains + β-propeller domains + an O-linked sugar domain + TM + CYT (LA1-2-Flag, residues 31–111 and 236–873), and LA6 + LA7 + LA8 + EGF-like domains + β-propeller domains + an O-linked sugar domain + TM + CYT (LA6-8-Flag, residues 236–873). The VLDLR-encoding vectors were packaged as Lentiviruses in HEK 293T cells with psPAX2 and pMD2.G. Then K562 cells were exposed to filtered (0.45 μm) supernatants containing lentivirus for 48 h. We selected transduced cell populations with puromycin (2 μg ml^−1^). For all constructs, we used a FACS sorting step to select positive cell subpopulations and confirmed construct expression in cell surface using Flag-tag antibody staining.

### Cell surface staining of receptors

Overexpressing cells were incubated in PBS containing 5% (v/v) goat serum (blocking buffer) for 30 min, followed by incubation with an anti-Flag antibody (Sigma, F7425), or no antibody in PBS containing 2% (v/v) goat serum (binding buffer) for 1 h. Following incubation, cells were washed three times in binding buffer and then incubated with Alexa Fluor 488-conjugated donkey anti-rabbit IgG (Thermo Fisher, R37118) at 1:2000 dilution for 30 min. After two washes, the cells were fixed with 2% (v/v) formalin, and cell surface receptor expression was detected by FACS using a Cytomics TM FC 500 (Beckman Coulter).

### Generation of SFV E1 mutants

The infectious cDNA clone of the SFV eGFP-reporter virus was used to create the E1 mutants. A QuickChange^TM^ site-directed mutagenesis kit (Invitrogen) was utilized to introduce mutations of selected E1 amino acid residues (E292A, N325A, G326S, D327A, K345A, K347A, and T348A) at the LA3 binding interface. The obtained mutant plasmids were sequenced to confirm the introduced mutations and the absence of other changes. Then, plasmids were transfected into BHK-21 cells using PEI transfection reagent (Sigma). The stock of rescued virus (P0 stock) was collected 60 h after transfection. To obtain P1 stock, 95% confluent BHK-21 cells grown on T75 flasks were infected with P0 stock at an MOI of 5. At 24 h post-infection, the supernatant (P1 stock) was collected, and stored at −80°C. All rescued viruses were sequenced and titrated by standard plaque assays.

### Cryo-EM sample preparation and data collection

To prepare the SFV-VLDLR LBD, SFV-LA3-5, SFV-LA3, and SFV-LA5 complexes, purified SFV particles were incubated with VLDLR_LBD_-Fc, LA3-5-Fc, LA3-Fc or LA5-Fc protein at a ratio of 1:240 or 1:30 at 4°C for 2 h, respectively. A 3.5 μL aliquot of the free-SFV virus, SFV-VLDLR_LBD_-Fc, SFV-LA3-5-Fc, SFV-LA3-Fc or SFV-LA5-Fc complex at pH 7.4 was applied on a freshly glow-discharged Au grid (Quantifol R1.2/1.3 Mu200). After a 5 s incubation for particle absorption, the excess buffer solution was blotted and the grid was plunge-frozen using a Vitrobot Mark IV operated at 4°C and 100% humidity.

Cryo-EM data were collected using a 300 kV Titan Krios (FEI) microscope equipped with a K2 summit camera (Gatan). Movies were recorded in super-resolution counting mode with a calibrated pixel size of 1.076 Å. Each exposure was performed with an accumulative dose of 50 e-/Å2, which was fractionated into 32 frames for each image stack. The final defocus range of the micrographs was -1.5 to -3.0 μm.

### Image processing

All the frame stacks were aligned with MotionCor2 to generate micrographs [41]. Parameters of these aligned micrographs were estimated using GCTF [42]. All subsequent data processing steps were conducted using RELION and Cryosparc [43,44]. For naked SFV virion, SFV in complex with LA3, and SFV in complex with LA5, a total of 2,167, 10,424, and 5,123 datasets were collected, respectively, and 39,346, 49,626, and 35,445 bin4 particles were automatically picked using RELION AutoPick and extracted from aligned micrographs, respectively. Relion 2D classification was used to select particles to reconstruct the icosahedral symmetric structures and all the complexes were reconstructed to the Nyquist limit of bin4 data. Furthermore, the block-based reconstruction algorithm was imposed to reconstruct the blocks, which contained the intact symmetric unit of the virion and complexes. These reconstruction steps were performed using local refinement imposed in CryoSPARC and resulted in density maps with resolutions of 3.02 Å, 3.45 Å, and 3.55 Å respectively. For SFV in complex with LA3-5 and intact VLDLR, 4,036 and 2,351 frame stacks were collected, and 52,869 and 40,701 particles were picked from aligned micrographs, respectively. After 2D Classification, 44,701 and 33,471 particles were selected to reconstruct complexes with icosahedral symmetry imposed and resulted in a resolution of 8.60 Å, the limit of Nyquist of bin4 data. The block-based reconstruction algorithm was imposed and the blocks containing the 2-fold axis region were re-extracted. The blocks of the 2-fold axis region of SFV in complex with LA3-5 were reconstructed with local refinement imposed in CryoSPARC and resulted in a density map with a resolution of 3.44 Å. In addition, two rounds of 3D classification were used for the blocks of the 2-fold axis region of SFV in complex with VLDLR to remove the LA3 binding-like blocks and the bias generated by the icosahedral symmetry of the virus. Finally, a total of 139,349 blocks were selected resulting in a density map with a resolution of 3.70 Å. All the resolutions were determined based on the gold-standard Fourier shell correlation (FSC) with 0.143 criterion, and the local resolution of final maps was estimated by ResMap [45].

### Model building and refinement

The model of the complex was built according to density maps reconstructed in this study. The cryo-EM structure of SFV VLP in complex with LA3 (PDB: 8IHP) and the structures of LA2, LA4, and LA5 predicted by AlphaFold2 were used as references. The models were combined and refined by Coot [46], and real space refinement imposed in PHENIX [47]. Both the structures of SFV in complex with LA3-5 and with VLDLR were built by fitting in the electron density and connected manually using Coot. The geometries of structures were validated using MolProbity [48], and refinement statics are shown in S1 Table.

### Quantification and statistical analyses

Statistical significance was assigned as p < 0.05 using GraphPad Prism Version 8.0. Experiments were analyzed by unpaired two-tailed t-tests with the Welch post-correction of one-way ANOVA with Tukey’s multiple comparisons test. The statistical tests, number of independent experiments, and number of experimental replicates are indicated in the Figure legends or relevant method section. K_D_ values for Surface plasmon resonance experiments were performed with Biacore 8K Evaluation Software 3.0 using a standard 1:1 binding model.

## Supporting information

S1 Fig Overexpression cell lines validation and Quantification analysis of different LA repeats of VLDLR-mediated SFV entry into cells. **(A)** Immunostaining to monitor cell surface receptor expression. Anti-FLAG staining of WT K562 cells or K562 cells expressing the indicated constructs as monitored by FACS. **(B)** Quantification analysis of GFP-expressing cells after SFV-eGFP infection. K562 cells expressing the indicated constructs in (A) or WT K562 cells were infected with eGFP-expressing SFV at MOI of 5 for 12 h. The cells were examined for virus infection (eGFP) using FACS.
(TIF)

S2 Fig Reconstructions of SFV virion, LA3-SFV and LA5-SFV. (**A**) Diagrams showing the workflow of data processing and reconstruction procedure of SFV version (left), LA3-SFV (middle), and LA5-SFV (right). (**B**) Gold standard FSCs of the reconstructions calculated with the asymmetric units block of SFV version (left), LA3-SFV (middle), and LA5-SFV (right). (**C**) Local resolution maps of SFV version (left), LA3-SFV (middle), and LA5-SFV (right). (**D**) Structure density of E1-DIII of SFV version (left), E1-DIII and its bound LA3 (middle), E1-DIII and its bound LA5 (right).
(TIF)

S3 Fig Structural analysis of LA repeats and receptor binding domain of SFV. (**A**) Sequence alignments of the eight LA repeats. The key contacting residues of LA3 are indicated with blue triangles at the top of the alignments. (**B**) Ribbon-rendered representation showing the superimposition of LA repeats of VLDLR. (**C**) Ribbon-rendered representation showing the model of LA3 bound fivefold axis region (left). The zoom-in view of a ribbon-rendered representation of the area boxed with a black dash line (right). The steric clashes are marked with yellow spheres and the transparent LA3 repeats have steric clashes with the other two LA3s. (**D**) Surface-rendered representation showing per residue R.M.S.D. map of LA3 bound SFV receptor binding domain and naked SFV virion. A higher R.M.S.D. value indicates greater variation. (**E**) The left top surface-rendered representation showing the structure of E1-DIII of SFV, and the LA3 binding site are colored orange. The other surface-rendered representations showing per residue R.M.S.D. map of site-1 comparing site-2, site-2 comparing site-3, and site-1 comparing to site-3. A higher R.M.S.D. value indicates greater variation.
(TIF)

S4 Fig Reconstructions of LA3-5-SFV and VLDLR-SFV. (**A**) Diagrams showing the workflow of data processing and reconstruction procedure of LA3-5-SFV (left), and VLDLR-SFV (right). (**B**) Gold standard FSCs of the reconstructions calculated with the twofold axis region block of LA3-5-SFV (left), and VLDLR-SFV (right). (**C**) Local resolution maps of LA3-5-SFV (left), and VLDLR-SFV (right). (**D**) Structure density of E1-DIII of LA3-5-SFV (top left), E1-DIII of VLDLR-SFV (top right), bound LA repeat of LA3-5-SFV (bottom left), and bound LA repeat of VLDLR-SFV (bottom right).
(TIF)

S1 Table Cryo-EM data collection, refinement and validation statistics, related to Fig 2 and Fig 6. (DOCX)
