# Supplementary figures and images for: Structural insights into Semiliki forest virus receptor binding modes indicate novel mechanism of virus endocytosis
Source: PLoS Pathog. 2024 Dec 20;20(12):e1012770. doi: 10.1371/journal.ppat.1012770 (PMC11661604; doi:10.1371/journal.ppat.1012770)

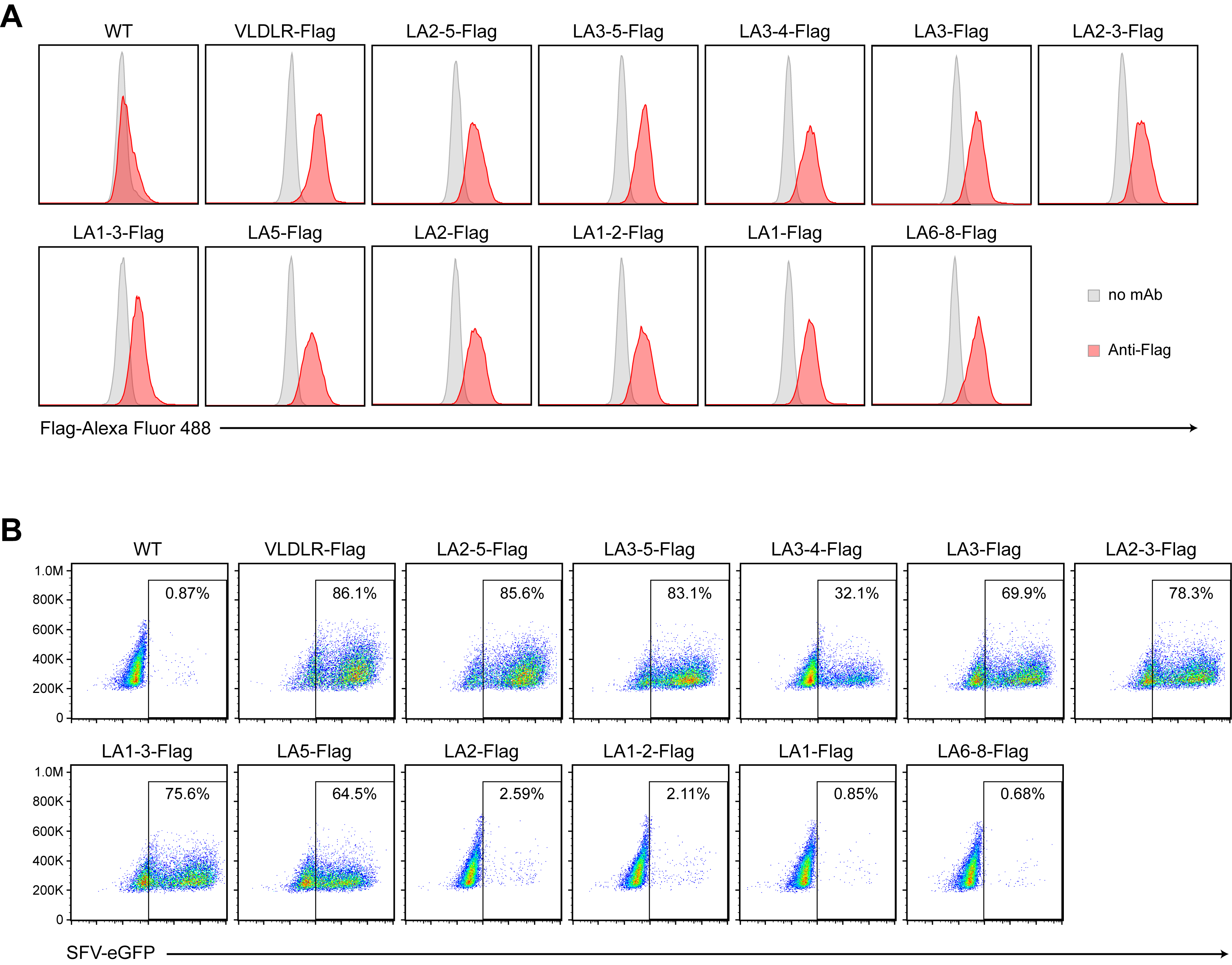

Supplement: S1 Fig — (A) Immunostaining to monitor cell surface receptor expression. Anti-FLAG staining of WT K562 cells or K562 cells expressing the indicated constructs as monitored by FACS. (B) Quantification analysis of GFP-expressing cells after SFV-eGFP infection. K562 cells expressing the indicated constructs in (A) or WT K562 cells were infected with eGFP-expressing SFV at MOI of 5 for 12 h. The cells were examined for virus infection (eGFP) using FACS. (TIF) [file ppat.1012770.s001.tif]

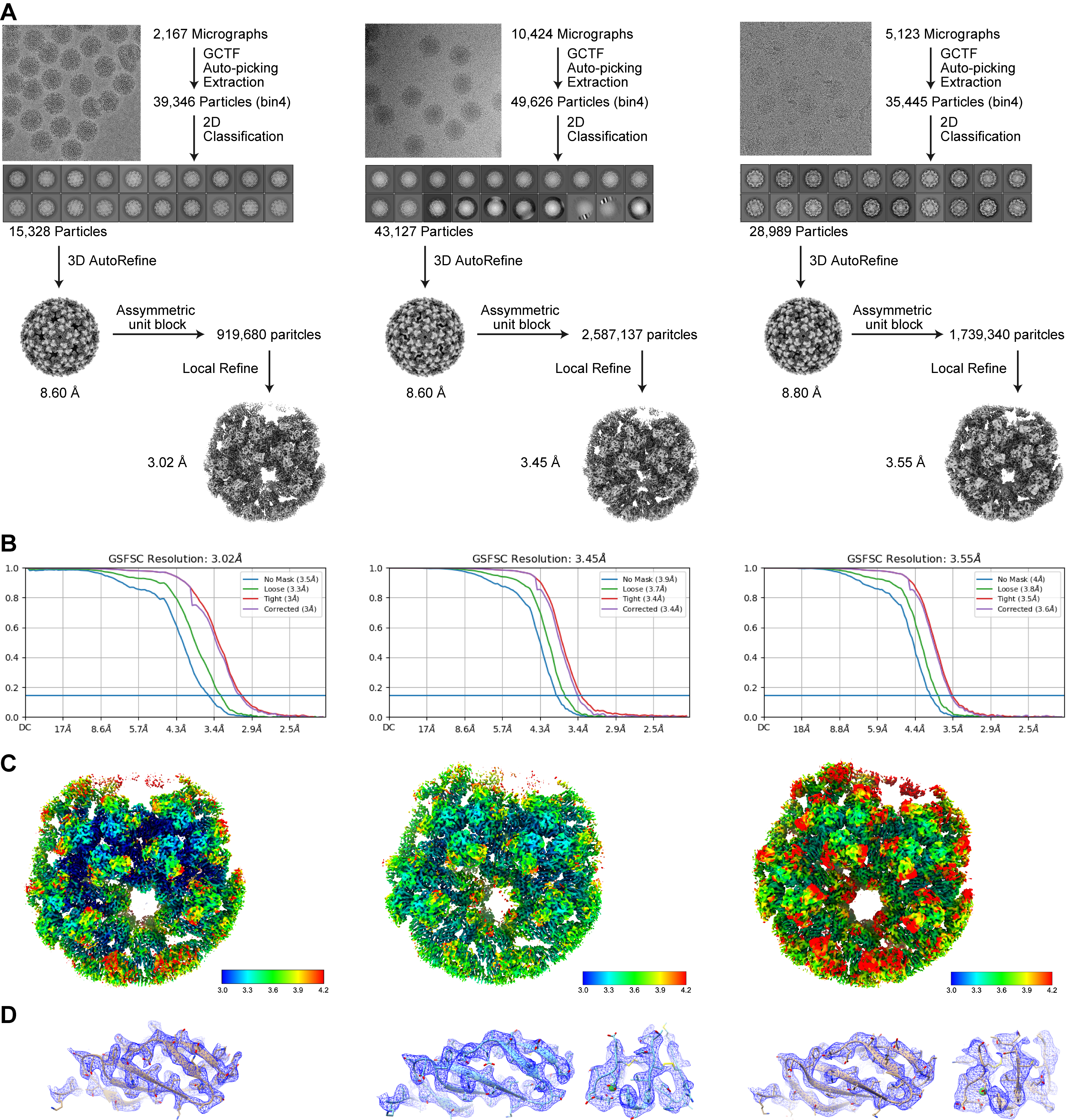

Supplement: S2 Fig — (A) Diagrams showing the workflow of data processing and reconstruction procedure of SFV version (left), LA3-SFV (middle), and LA5-SFV (right). (B) Gold standard FSCs of the reconstructions calculated with the asymmetric units block of SFV version (left), LA3-SFV (middle), and LA5-SFV (right). (C) Local resolution maps of SFV version (left), LA3-SFV (middle), and LA5-SFV (right). (D) Structure density of E1-DIII of SFV version (left), E1-DIII and its bound LA3 (middle), E1-DIII and its bound LA5 (right). (TIF) [file ppat.1012770.s002.tif]

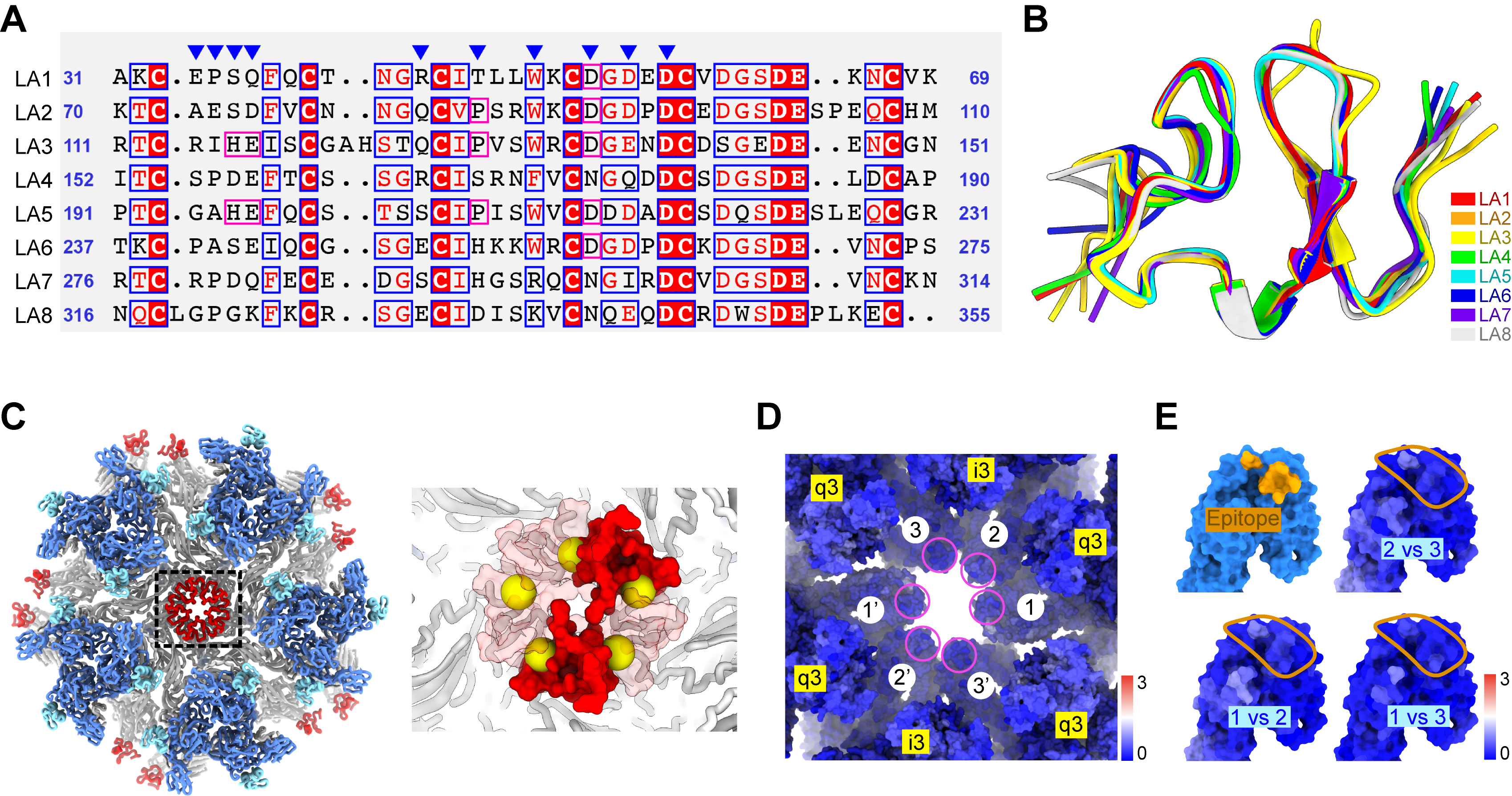

Supplement: S3 Fig — (A) Sequence alignments of the eight LA repeats. The key contacting residues of LA3 are indicated with blue triangles at the top of the alignments. (B) Ribbon-rendered representation showing the superimposition of LA repeats of VLDLR. (C) Ribbon-rendered representation showing the model of LA3 bound fivefold axis region (left). The zoom-in view of a ribbon-rendered representation of the area boxed with a black dash line (right). The steric clashes are marked with yellow spheres and the transparent LA3 repeats have steric clashes with the other two LA3s. (D) Surface-rendered representation showing per residue R.M.S.D. map of LA3 bound SFV receptor binding domain and naked SFV virion. A higher R.M.S.D. value indicates greater variation. (E) The left top surface-rendered representation showing the structure of E1-DIII of SFV, and the LA3 binding site are colored orange. The other surface-rendered representations showing per residue R.M.S.D. map of site-1 comparing site-2, site-2 comparing site-3, and site-1 comparing to site-3. A higher R.M.S.D. value indicates greater variation. (TIF) [file ppat.1012770.s003.tif]

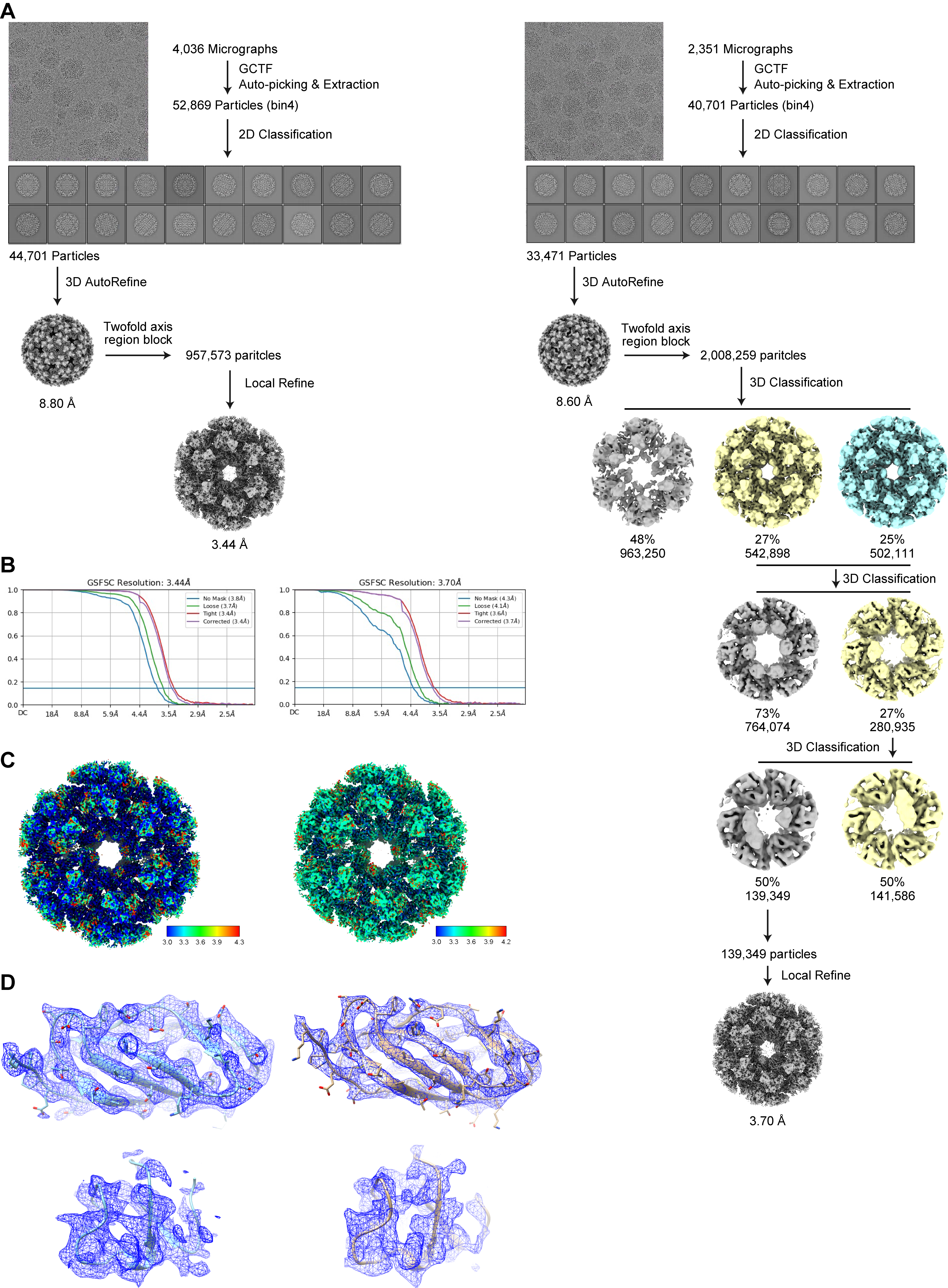

Supplement: S4 Fig — (A) Diagrams showing the workflow of data processing and reconstruction procedure of LA3-5-SFV (left), and VLDLR-SFV (right). (B) Gold standard FSCs of the reconstructions calculated with the twofold axis region block of LA3-5-SFV (left), and VLDLR-SFV (right). (C) Local resolution maps of LA3-5-SFV (left), and VLDLR-SFV (right). (D) Structure density of E1-DIII of LA3-5-SFV (top left), E1-DIII of VLDLR-SFV (top right), bound LA repeat of LA3-5-SFV (bottom left), and bound LA repeat of VLDLR-SFV (bottom right). (TIF) [file ppat.1012770.s004.tif]
