# Supplementary material for: Structural insights into Semiliki forest virus receptor binding modes indicate novel mechanism of virus endocytosis
Source: PLoS Pathog. 2024 Dec 20;20(12):e1012770. doi: 10.1371/journal.ppat.1012770 (PMC11661604; doi:10.1371/journal.ppat.1012770)
Supplement: S1 Table — (DOCX) [file ppat.1012770.s005.docx]

**S1 Table. Cryo-EM data collection, refinement and validation statistics, related to Figure 2 and Figure 6.**

|  | SFV virion | SFV-LA3 | SFV-LA5 | SFV-LA3-5 | SFV-VLDLR |
| --- | --- | --- | --- | --- | --- |
| **Data collection and processing** | | | | | |
| Magnification | 130,000 | | | | |
| Voltage (KV) | 300 | | | | |
| Electron exposure (e-/ Å^2­­^) | 50 | | | | |
| Defocus range (μm) | 1.5 - 3 | | | | |
| Pixel size (Å) | 1.076 | 1.076 | 1.065 | 1.1 | 1.1 |
| Micrographs (no.) | 2,167 | 10,424 | 5,123 | 4,036 | 2,351 |
| Symmetric imposed | Icosahedral | | | | |
| Initial particle image (no.) | 39,346 | 49,626 | 35,445 | 52,869 | 40,701 |
| Final particle image (no.) | 15,328 | 43,127 | 28,989 | 44,701 | 33,471 |
| **Block based refinement** | **Asymmetric unit** | **Asymmetric unit** | **Asymmetric unit** | **Twofold axis region** | **Twofold axis region** |
| Final particle images (no.) | 919,680 | 2,587,137 | 1,739,340 | 957,573 | 139,349 |
| Map resolution (no.) | 3.02 | 3.45 | 3.55 | 3.44 | 3.70 |
| FSC threshold | 0.143 | | | | |
| **Model refinement** |  |  |  |  |  |
| Initial model | 8IHP | | | | |
| Model resolution (Å) | 3.2 | 3.6 | 3.7 | 3.7 | 3.9 |
| Model resolution range (Å) | 2.9-3.2 | 3.3-3.6 | 3.4-3.7 | 3.2-3.6 | 3.4-3.9 |
| Map sharpening B factor (Å^2^) | 96.1 | 163.1 | 145.8 | 159.9 | 129.7 |
| **Model composition** |  | | | | |
| Non-hydrogen atoms | 33,424 | 37,740 | 34,264 | 67,744 | 134,901 |
| Protein residues | 4,276 | 4,452 | 4,396 | 86,73 | 18,286 |
| Ligands | 36 | 40 | 36 | 72 | 144 |
| **B factors (Å^2^)** |  | | | | |
| Protein | 93.36 | 57.08 | 90.51 | 80.25 | 73.41 |
| Ligand | 148.16 | 97.72 | 119.48 | 143.86 | 133.22 |
| **R.m.s. deviations** |  | | | | |
| Bonds length (Å) | 0.008 | 0.003 | 0.003 | 0.004 | 0.007 |
| Bond angles (°) | 0.918 | 0.581 | 0.599 | 0.648 | 0.795 |
| **Validation** |  | | | | |
| MolProbity score | 1.90 | 1.8 | 1.74 | 1.82 | 2.02 |
| Clashscore | 8.22 | 6.36 | 5.86 | 7.03 | 10.10 |
| Poor rotamers (%) | 0.003 | 0.08 | 0.003 | 0.00 | 0.03 |
| **Ramachandran plot** |  | | | | |
| Favored (%) | 92.81 | 92.93 | 93.58 | 93.45 | 91.76 |
| Allowed (%) | 6.95 | 6.87 | 6.20 | 6.34 | 7.97 |
| Disallowed (%) | 0.24 | 0.20 | 0.23 | 0.21 | 0.27 |
